# Evaluation of determinants of the serological response to the quadrivalent split‐inactivated influenza vaccine

**DOI:** 10.15252/msb.202110724

**Published:** 2022-05-06

**Authors:** Shaohuan Wu, Ted M Ross, Michael A Carlock, Elodie Ghedin, Hyungwon Choi, Christine Vogel

**Affiliations:** ^1^ Center for Genomics and Systems Biology New York University NY USA; ^2^ Department of Infectious Diseases College of Veterinary Medicine University of Georgia Athens GA USA; ^3^ Center for Vaccines and Immunology University of Georgia Athens GA USA; ^4^ Systems Genomics Section Laboratory of Parasitic Diseases NIAID, NIH Bethesda MD USA; ^5^ Department of Medicine Yong Loo Lin School of Medicine National University of Singapore Singapore City Singapore

**Keywords:** human cohort, immune response, influenza, split‐inactivated influenza vaccine, statistical modeling, Computational Biology, Immunology, Microbiology, Virology & Host Pathogen Interaction

## Abstract

The seasonal influenza vaccine is only effective in half of the vaccinated population. To identify determinants of vaccine efficacy, we used data from > 1,300 vaccination events to predict the response to vaccination measured as seroconversion as well as hemagglutination inhibition (HAI) titer levels one year after. We evaluated the predictive capabilities of age, body mass index (BMI), sex, race, comorbidities, vaccination history, and baseline HAI titers, as well as vaccination month and vaccine dose in multiple linear regression models. The models predicted the categorical response for > 75% of the cases in all subsets with one exception. Prior vaccination, baseline titer level, and age were the major determinants of seroconversion, all of which had negative effects. Further, we identified a gender effect in older participants and an effect of vaccination month. BMI had a surprisingly small effect, likely due to its correlation with age. Comorbidities, vaccine dose, and race had negligible effects. Our models can generate a new seroconversion score that is corrected for the impact of these factors which can facilitate future biomarker identification.

## Graphical abstract

Computational modeling quantifies the effects of confounding factors on the serological response to flu vaccination in large human cohorts and reveals a differential impact of prior vaccination status, recipient age, and the month of vaccination.

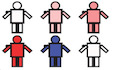

## Introduction

Influenza virus infections represent a continuous threat to public health, as vaccine effectiveness is typically low, ranging from 19 to 60% during the 2009 to 2018 seasons in the United States, according to the Center for Disease Control (https://www.cdc.gov/flu). The widely used split‐inactivated influenza vaccine is typically quadrivalent with an antigen for all four virus subtypes, H1N1 and H3N2 (Influenza A) subtypes, and Yamagata and Victoria lineages (Influenza B). Understanding predictors of vaccine efficacy is an ongoing public health challenge.

While antigenic drift and shift caused by frequent mutations in circulating viral strains are long‐known influencers of vaccine efficacy, there is increasing recognition of other factors intrinsic to the human host which impact vaccine efficacy and/or severity of an influenza infection. These factors can be genetic (Franco *et al*, 2013; Orrù *et al*, 2013; Brodin *et al*, 2015), epigenetic (Zimmermann *et al*, 2016), or represent pre‐existing immunity (Voth *et al*, 1966; Beyer *et al*, 1996; Henn *et al*, 2013; HIPC‐CHI Signatures Project Team & HIPC‐I Consortium, 2017) as caused by prior infection or vaccination (Zost *et al*, 2017; Gouma *et al*, 2020; Sung *et al*, 2021). Further, demographic factors such as age (Goodwin *et al*, 2006; Henry *et al*, 2019; Henry *et al*, 2019), obesity (Honce & Schultz‐Cherry, 2019; Honce *et al*, 2020), and sex (Klein & Flanagan, 2016; Fink *et al*, 2018; Voigt *et al*, 2019) are thought to play a role. In addition, recent studies have shown that vaccination time during a flu season can also affect the response to the vaccine (Penkert *et al*, 2021). However, many of these factors are intercorrelated, for example, prior vaccination and baseline antibody titer level as well as age, obesity, and other comorbidities. Most existing studies examined the effect of one or a few of these factors (Goodwin *et al*, 2006; Gouma *et al*, 2020; Penkert *et al*, 2021; Sung *et al*, 2021) without comprehensive integration.

Here, we addressed this gap in knowledge by using a large cohort dataset to construct multiple linear regression models that predicted the response to the split‐inactivated influenza vaccine based on nine variables known for participants. We performed the prediction separately in three different age groups for both seroconversion after 3–4 weeks and antibody titer levels in the subsequent year. We evaluated the impact of each individual factor adjusting for the effects of the remaining factors.

## Results

### Mining a large cohort study with > 1,300 vaccination events

**Figure 1:**
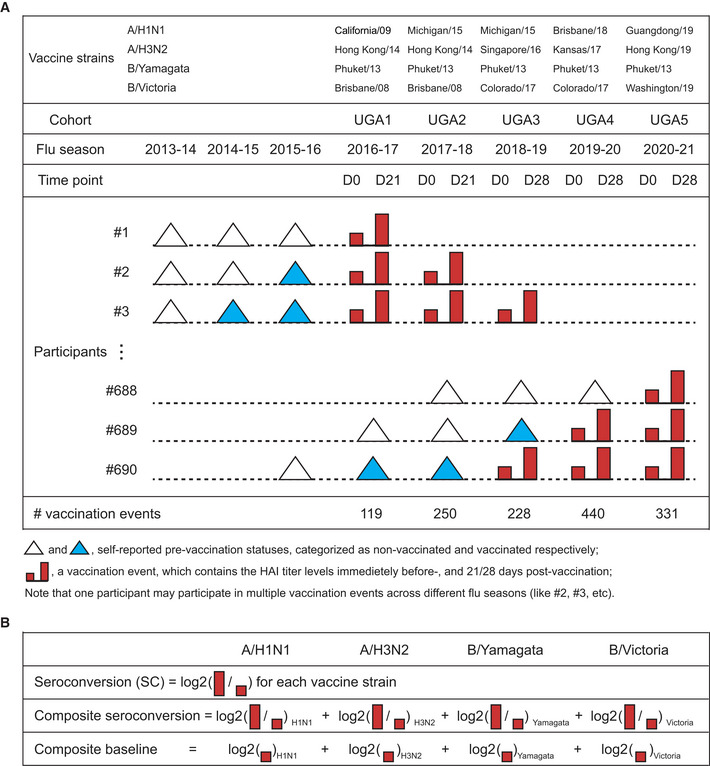
Illustration of the UGA cohort study Graphical illustration of the 1,368 vaccination events recorded for the 690 participants, with the self‐reported vaccination history for three years prior to joining the study. Many participants returned for several years. The table also lists the vaccine strains used as well as the cohort label (UGA1‐UGA5). Titer levels measured at Day 0 (D0) are referred to as baseline for the respective season. Data recorded as vaccination events from cohorts UGA1‐3 and UGA5 were used for model training; data from UGA4 were used for evaluation of the predictions.Graphical definitions of seroconversion, composite seroconversion, and composite baseline used in the study. Seroconversion is unitless. Baseline titer levels refer to HAI titer levels.

We used one of the largest cohort studies available, involving ~ 700 participants monitored over five flu seasons (cohorts), producing 1,368 vaccination events (Fig 1A). A vaccination event is defined as a participant receiving the flu vaccine in a specific season, with HAI (hemagglutination inhibition) titer levels against the vaccine strains being assessed on day 0 and day 21/28. We predicted both seroconversion (*Seroconversion*), measured as the log_2_ ratio between HAI titer levels against the four vaccine strains 3 or 4 weeks post‐vaccination (D21 or D28, respectively) and HAI titer at D0, and the baseline (BL, D0) HAI titer levels in the subsequent year (*BaselineSY*) (Fig 1B). In addition to demographic factors, such as age, body mass index (BMI), sex, race, comorbidities, and vaccination history of participants, we included month of vaccination (September to February) and vaccine dose (for participants ≥ 65 years old) in the prediction. Based on the long‐lasting effect of vaccination (Appendix Fig S1), we defined participants as prevaccinated if they had received the vaccine in the year prior to joining the study, and as naive if they had had no vaccine within the last 3 years prior to joining the study, removing cases with a mixed vaccination history.

**Figure 2:**
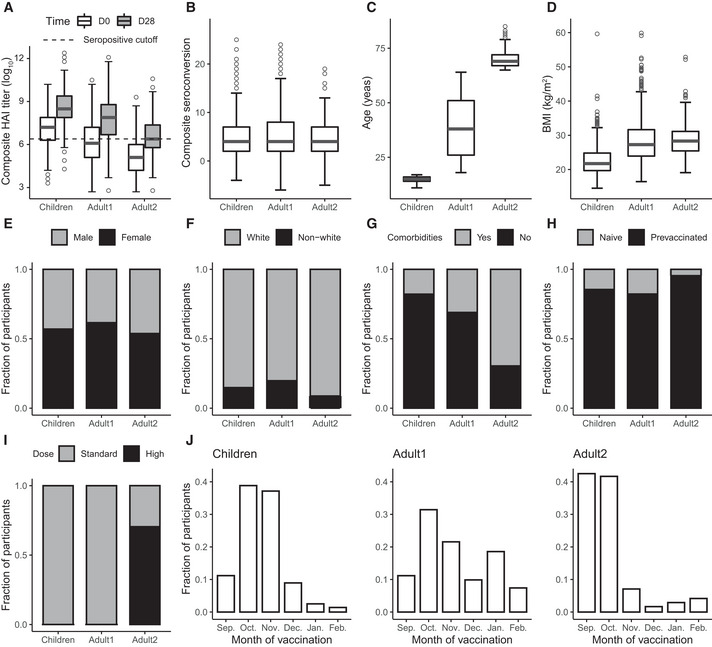
Overview of the datasets for three subpopulations ADistribution of composite D0 and D28 HAI titer levels in different subpopulations: *Children* (< 18 years old), *Adult1* (18‐64 years old), and *Adult2* (≥ 65 years old) which comprise 358, 770, and 240 total data points (vaccination events) across all five cohorts UGA1‐5, respectively. Similar to the definition of composite seroconversion, composite D0 and D28 titer levels are defined as the sum of log_2_(D0 titer level) or sum of log_2_(D28 titer level) across 4 vaccine strains, respectively. Seropositivity cutoff is the composite titer level at a titer larger than 40 in all 4 strains (4*log_2_(40)). HAI, hemagglutination inhibition assay.B–DDistribution of composite seroconversion, age, and BMI in three subpopulations across all five cohorts UGA1‐5. BMI, body mass index.E–IDistribution of categorical variables in three subpopulations across all five cohorts UGA1‐5. For the comorbidities prior, *yes* indicates having at least one of the comorbidities that are surveyed and *no* indicates having none. (G). For vaccine dose, high dose is offered as an option only to *Adult2* subpopulation (I).JFraction of participants that are vaccinated in each month in a flu season across all 5 cohorts UGA1‐5, in *Children* (left), *Adult1* (middle), and *Adult2* (right) subpopulations. In the box and whiskers plots in (A–D), the central band represents the median, the lower and upper hinges represent 25^th^ and 75^th^ quantiles respectively, the lower and upper flat arrows represent extreme values that are within 1.5*IQR (internal‐quantile range) from the lower and upper hinges respectively, and the empty circles represent outliers, for example, extreme values that are beyond 1.5*IQR from the hinges. There are 358, 770, and 240 vaccination events (data points) in *Children*, *Adult1*, and *Adult2* subpopulations, respectively.

We then separated participants into three subpopulations: *Children* (< 18 years), *Adult1* (18–64 years), and *Adult2* (≥ 65 years). Fig 2 shows the distributions of variables across these three groups. Most participants across all groups were prevaccinated which substantially affects baseline HAI levels (Fig 2H). However, vaccination in the previous year was not the only determinant of the baseline HAI titer level (as discussed below), emphasizing the need for predictive modeling of both factors. For example, despite prior vaccination of most participants, the average baseline HAI level was lower in the *Adult2* group compared to the other two groups (Fig 2A), illustrating the need for continuous vaccination in this age group as well as an improved understanding of factors that impact successful response to vaccination. In addition, despite the substantial age differences, seroconversion was on average similar across three age groups (Fig 2B and C), further supporting the hypothesis that factors other than age determine the serological response.

Fig 2D–J show the distributions of other variables in the three subpopulations. *Adult1* and *Adult2* had on average higher BMIs than *Children*, and most *Adult2* participants had one or more comorbidities (Fig 2D and G). The three subpopulations were similar with respect to distributions of the remaining demographic factors, that is, sex and race (Fig 2E and F). Most of the participants were white. Only *Adult2* participants had been offered the high dose of the vaccine (Fig 2I). Finally, *Children* and *Adult2* participants had primarily been vaccinated in the first three months of a flu season while *Adult1* participants were vaccinated relatively evenly throughout the season (Fig 2J). For these reasons, dose and vaccination month were only useful predictors in the *Adult2* and *Adult1* subpopulations, respectively (see below).

### Predicting the vaccine response

**Figure 3:**
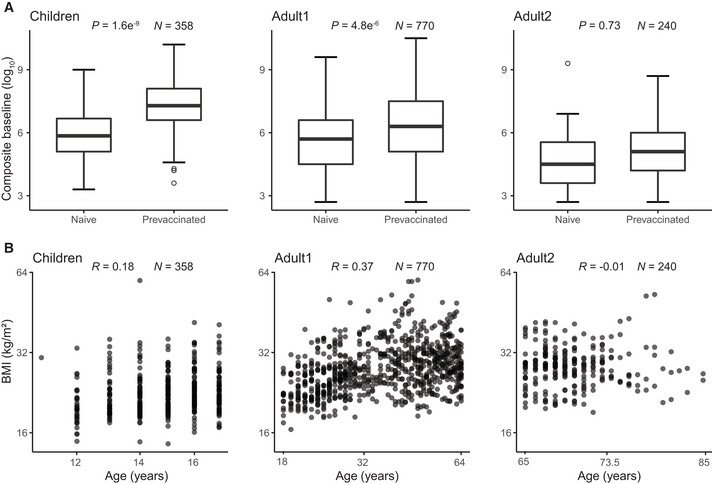
Relationships between some of the priors Correlation between vaccination history and baseline across three subpopulations across all five cohorts UGA1‐5. The central band represents the median, the lower and upper hinges represent 25^th^ and 75^th^ quantiles respectively, the lower and upper flat arrows represent extreme values that are within 1.5*IQR (internal‐quantile range) from the lower and upper hinges respectively, and the empty circles represent outliers, for example, extreme values that are beyond 1.5*IQR from the hinges. *N* is the number of vaccination events. *P* value is calculated from a *T* test.Correlation between age and BMI across three subpopulations across all five cohorts UGA1‐5. *R* is calculated from Pearson’s correlation. BMI, body mass index.

Several variables showed some correlation with each other, highlighting the need to model the effect of each variable while controlling for the impact of the other variables. For example, prevaccinated participants had higher baseline titer levels than naive participants, as mentioned above (Fig 3A), and age and BMI are also positively correlated (Fig 3B). However, Fig 3A also shows that prior vaccination only partially predicts the baseline titer level due to the impact of additional factors which our study aimed to identify.

**Figure 4:**
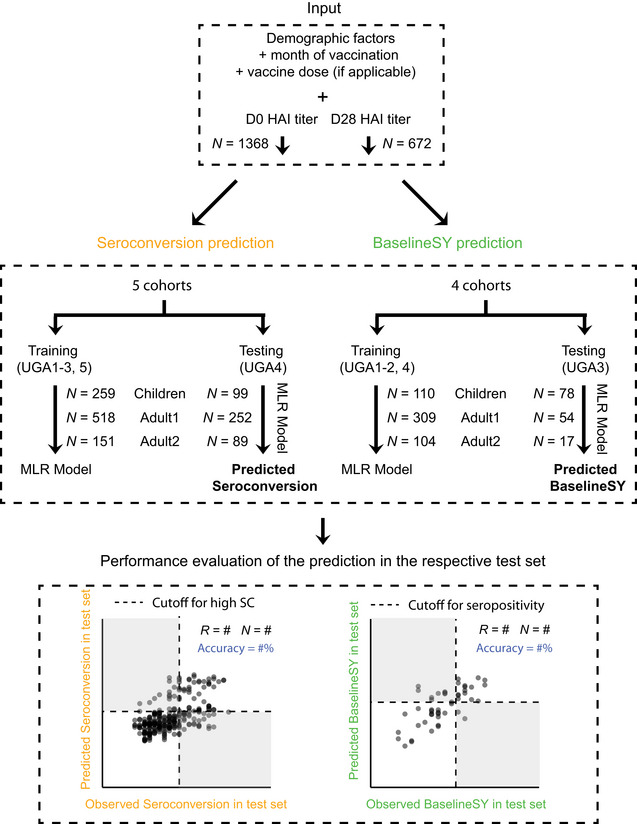
The computational framework used to predict the serological response to flu vaccination Variables for *Seroconversion* prediction and *BaselineSY* prediction, respectively, in each of the three subpopulations, and machine learning strategy used to predict *Seroconversion* and *BaselineSY*. We used two metrics to evaluate the performance of the models: correlation coefficient (*R*) and accuracy (%) of values observed in the test dataset (UGA4) compared to values predicted for the test dataset (UGA4) using the models trained on UGA1‐3 and UGA5. Accuracy describes the extent to which we correctly predicted the category. Cutoffs for high seroconversion and seropositivity are the same as those in Fig 2. Dashed lines mark the cutoff for high seroconversion (≥ 8) or seropositivity (≥ 4*log_2_(40)), respectively. White and grey quadrants show areas of correct and incorrect predictions respectively, used to calculate accuracy. All modeling, predicting, and evaluation have been done for the three subpopulations separately using UGA1‐⅗ for training and UGA4 for testing. Percentages listed in blue denote correct predictions (accuracy). MLR, multiple linear regression.

We predicted *Seroconversion* and *BaselineSY* for both the four vaccine strains individually and the composite value calculated as the sum of the values across four strains (Fig 4). For each prediction, we trained the model on four cohorts (three for *BaselineSY*) and evaluated it on an independent fifth cohort (fourth cohort for *BaselineSY*), as detailed in the Materials and Methods. The evaluation cohort was not used in the model training and was chosen based on appropriate data distributions (Materials and Methods). The models obtained from the training sets are listed in Dataset EV1. Appendix Fig S3A and B show the results when evaluating the models across the entire dataset.

**Figure 5:**
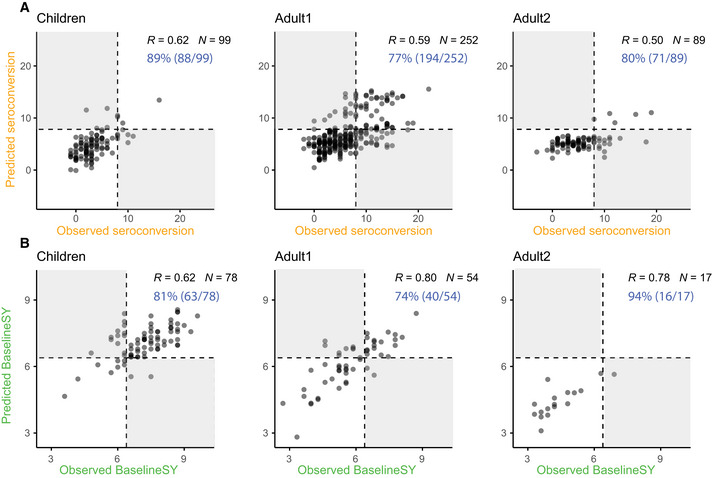
Prediction accuracies for *Seroconversion* and *BaselineSY* in three subpopulations Observed vs. predicted values for *Seroconversion*. Predicted values are obtained for the independent test set only (UGA4, see Fig 1), not the entire dataset. Results for the entire subpopulations are shown in Appendix Fig S3; results for a randomly chosen test dataset are shown in Appendix Fig S5B. Dashed lines mark the cutoff for high seroconversion (≥ 8). White and grey quadrants show areas of correct and incorrect prediction of high and low/no seroconversion, respectively. Percentages listed in blue denote correct predictions.Observed vs. predicted values for *BaselineSY*. Predicted values are obtained for the independent test set only (predicting the baseline in the subsequent year for participants in the UGA3 cohort). Dashed lines mark the cutoff for seropositivity (≥ 4*log_2_(40)). White and grey quadrants show areas of correct and incorrect predictions respectively. Percentages listed in blue denote correct categorical predictions (accuracy). *R* is calculated from Pearson’s correlation.

We evaluated the models in two ways: (i) using the correlation (*R*) between predicted and observed values; and (ii) using the fraction of cases for which we correctly predicted the category of the response, that is, high vs. low *Seroconversion*; seropositive vs. negative *BaselineSY*, denoted as accuracy in Fig 5A and B. We predicted *Seroconversion* and *BaselineSY* accurately at 74% or higher in independent test sets for all three subpopulations. The models performed best with respect to prediction of *BaselineSY* for the *Adult2* subpopulation (≥65 years old) (94% accuracy). The models performed worst for prediction of *Seroconversion* and *BaselineSY* for the *Adult1* subpopulation (18–64 years old), indicating that for these participants, additional factors which were not considered in the model impacted the response to vaccination, such as specific comorbidities. As Appendix Table S3 shows, available data are currently too small to model the impact of specific comorbidities. Appendix Fig S5B shows the results for training and testing when evaluating on a randomly chosen test set which was left out from training. The correlation (*R*) between predicted and observed values was higher in all three subpopulations and the accuracy is also higher in *Children* and *Adult1* for the randomly chosen test set than what we observed when evaluating with one of the cohorts. The result suggests that evaluation with the independent cohort is a conservative approach.

Next, we explored the ability to predict *Seroconversion* without knowing the baseline titer level, to mimic a more practical scenario in which a vaccine recipient’s response is predicted without the need to draw blood and measure HAI titer levels. When repeating the modeling as before with all priors but baseline titer levels, the prediction accuracy was very similar across all three subpopulations compared to the original prediction (Appendix Fig S4), implying that we can still predict the overall response (high vs. low/none) for most participants. However, the models’ ability to predict actual *Seroconversion* level (i.e. *R* with the observed values) dropped.

Further, we tested if a participant being evaluated in the study over several seasons biased our results. We did not observe such bias, based on the following results. First, when comparing prediction outcomes between the set of one‐time participants and a set of randomly chosen vaccination events, we observed similar prediction accuracy (Appendix Fig S5A and B). Second, prediction results were also similar for the subset of entries evaluating the first time a person participated in the study versus the second time the person participated (subsequent season) (Appendix Fig S6A and B). The results indicate that the use of vaccination events as units in our model was valid.

### Identifying major predictors of *Seroconversion* and *BaselineSY*

**Figure 6:**
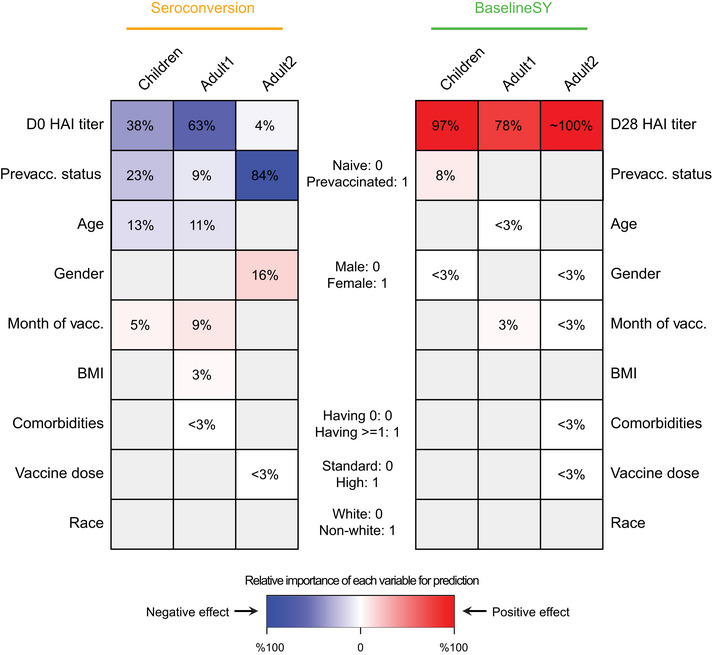
Contributions of individual priors to the response prediction Relative importance of each variable for prediction. The intensity of the color in each cell denotes the importance of the variable, measured as percentage of drop in prediction accuracy measured as squared correlation (R^2^) between observed and predicted values when leaving the variable (prior) out compared to the complete model (see Materials and Methods). Grey denotes variables not selected for modeling. The color denotes the direction of the correlation between the variable and the predicted value, that is, red and blue values denote positive and negative correlation, respectively, between the variable and *Seroconversion* or *BaselineSY*. For example, prevaccination status predicts both *Seroconversion* and *BaselineSY*; prevaccinated participants show lower (blue) *Seroconversion* (all subpopulations) and higher (red) *BaselineSY* (*Children* only). White denotes variables with importance ≤ 3% or with a coefficient ≤ 0.1 in absolute value. HAI, hemagglutination inhibition assay; vacc., vaccination. Appendix Table S1 contains the data underlying this figure.

**Figure 7:**
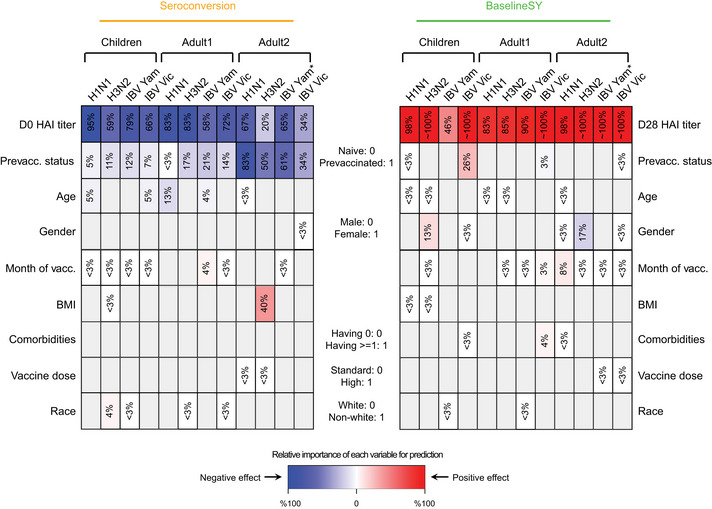
Contributions of individual priors to the response prediction in each vaccine strain Relative importance of each variable for prediction. The intensity of the color in each cell denotes the importance of different priors, measured as percentage of drop in prediction accuracy measured as squared correlation (R^2^) between observed and predicted values when leaving the prior out compared to the complete model (see Materials and Methods). Grey denotes variables not selected for the modeling. The color denotes the direction of the correlation between the variable and the predicted value, that is, red and blue values denote positive and negative correlation, respectively, between the variable and Seroconversion or BaselineSY. White denotes variables with importance ≤ 3% or with a coefficient ≤ 0.1 in absolute value. IBV, Influenza B Virus; HAI, influenza hemagglutination inhibition assay; vacc., vaccination; Vic, Victoria; Yam, Yamagata. *IBV Yamagata strain is absent in the vaccine in UGA cohorts 1–4, but present in UGA5. Appendix Table S2 contains the data underlying this figure.

Next, we estimated the relative importance of each variable in predicting *Seroconversion* and *BaselineSY* for both composite scores (Fig 6) and individual strains (Fig 7). Importantly, even though several variables are intercorrelated (Appendix Fig S2), our results show the *independent* effect of each variable. Appendix Tables S2 and S3 contain the data underlying Figs 6 and 7, respectively.

As expected, prior vaccination and high HAI baseline levels explained the majority of variation in *Seroconversion* in each subpopulation (Figs 6 and 7). Conversely, HAI titer levels at Day 21 or 28, but not vaccination history, predicted the baseline titer level in the subsequent year (*BaselineSY*). The exception was *Children* in which prior vaccination had a modestly positive impact on the vaccine longevity.

One exception was baseline HAI levels against the H3N2 strain which exhibited less predictive value for *Seroconversion* for the *Adult2* subpopulation (Fig 7). This might partially be explained by the type of A/H3N2 vaccine strain used in the UGA4 cohort, which served as the test set, compared to the other cohorts, which served as training sets (Fig 1A). The A/H3N2 vaccine strain in UGA4 originated from a different clade (3C.3a) compared to those used in previous years and therefore may have led to low baseline HAI titer levels.

While including such strain‐specific effects might improve future modeling results, we also showed that overall the “generic” strain information we used was a valid simplification: HAI titer levels against the UGA4 A/H3N2 strain (A/Kansas/2017) correlated well with those against the A/H3N2 vaccine strains used in UGA1‐3 (A/Hong Kong/2014 and A/Singapore/2016) (Appendix Fig S7B); HAI titer levels against the A/H1N1 vaccine strain used in UGA 4 (A/Brisbane/2018) also correlated well with the A/H1N1 strains used in UGA1‐3 (A/California/2009 and A/Michigan/2015) (Appendix Fig S7A)—indicating substantial cross‐reactivity of antibodies formed.

**Figure 8:**
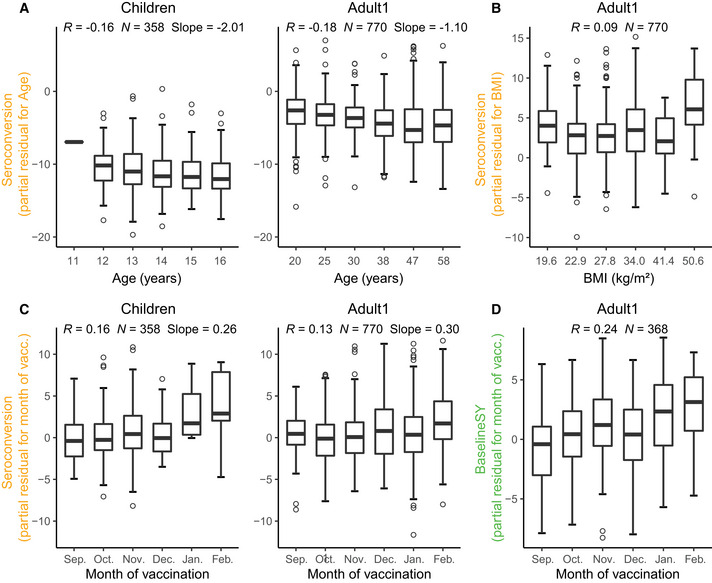
Independent relationships between age, BMI, and vaccination month, and the response to vaccination A–DPartial residual plots illustrating the relationship between a variable (x‐axis) and the target value after removing the impact of all other variables (partial residual, y‐axis), using data from all 5 cohorts UGA1‐5. Target values are either *Seroconversion* (A–C) or *BaselineSY* (D). We grouped variables along the *x*‐axis in (A–B) into 6 equally spaced bins, showing box and whiskers plots for each bin. The central band represents the median, the lower and upper hinges represent 25^th^ and 75^th^ quantiles respectively, the lower and upper flat arrows represent extreme values that are within 1.5*IQR (internal‐quantile range) from the lower and upper hinges, respectively, and the empty circles represent outliers, for example, extreme values that are beyond 1.5*IQR from the hinges. *N* is the number of vaccination events. The *R* values are calculated based on all data per subpopulation from Pearson’s correlation. Vacc., vaccination.

Unexpectedly, we observed an age effect among *Children* similar to that amongst the *Adult1* subpopulation (Figs 6 and 7): Older children had lower *Seroconversion* than younger children, in particular driven by the effect of the H1N1 strain. This negative relationship was weak, but statistically significant, as illustrated in Fig 8A which depicts the relationship between the variable and the predicted value removing the effects of all other variables. The relationship implies that immunosenescence starts before adulthood. In comparison, participant age did not predict longevity of the antibodies with respect to *BaselineSY*.

Another major predictor was the month of vaccination: the later children and adults < 65 years received the vaccine during the season (September to February), the higher the *Seroconversion* (Figs 6 and 8C). For the *Adult1* subpopulation, this positive effect might have been mostly driven by the effect of the IBV Yamagata strain (Fig 7). To examine whether this result was caused by the widespread circulation of the IBV Yamagata lineage viruses in 2017–2018, we examined information on circulating strains in the five flu seasons. We found that, indeed, B/Yamagata was the dominant lineage in 2017–2018 flu season, albeit still less prevalent than A subtypes. In three of the other four seasons, B/Yamagata was less prevalent than B/Victoria. Therefore, the types of circulating strains could not explain the significant effect of vaccination month observed for B/Yamagata in Adult1 subpopulation. The model could not evaluate this variable for the *Adult2* subpopulation due to the above‐mentioned bias with respect to vaccination month (Fig 2J). Similarly, the later adults younger than 65 years received the vaccine during the season, the better the long‐term response was, that is, with respect to antibody titers a year later (Fig 8D).

The result is consistent with a recent finding on the link to natural influenza infections that occur more frequently later in the season (Penkert *et al*, 2021). Such infections could cause false‐positive seroconversion (i.e., unrelated to vaccination) if they occur during or shortly after the participant is vaccinated. However, when restricting the data for the *Adult1* subpopulation to only those vaccination events from the first three months of the flu season, that is, mimicking the distributions for *Children* and *Adult2*, we did not observe improvement in prediction accuracy (Appendix Fig S8). Therefore, the wider range of vaccination months could not explain the decreased prediction ability in the *Adult1* subpopulation compared to that in *Children* and *Adult2* subpopulations. Other factors not considered in the model likely have an effect on the vaccine response in *Adult1*.

Surprisingly, BMI had only a minor influence on *Seroconversion* and no impact on *BaselineSY*: Only in the *Adult1* subpopulation, we observed a slightly positive effect (Figs 6 and 8B). Among individual strains, BMI had a positive impact on the response to the H3N2 component in the *Adult2* subpopulation. These results appear to contrast the known negative impact of obesity on influenza severity (Honce & Schultz‐Cherry, 2019; Honce *et al*, 2020). We speculate that the strong age effect masks the effect of BMI on *Seroconversion* in this study since BMI and age are positively correlated (Fig 3B). In other words, most of the potential BMI effect on vaccine efficacy in the models is already accounted for by the age effect; once age is considered, there is little to no additional contribution by knowing the BMI of a vaccine recipient. Future work might explore the additional, weaker non‐linear relationship between BMI and age (Fig 3B) which our models did not capture. In addition, future work might include measures of metabolic health other than BMI which were used in the related studies mentioned above and might contribute to the different results observed here.

Further, we found that women had higher *Seroconversion* than men in the *Adult2* subpopulation, largely driven by the IBV Victoria strain (Figs 6 and 7). Race had no effect on both *Seroconversion* and *BaselineSY*. Similarly, use of the high‐dose vaccine over the standard dose in the *Adult2* subpopulation had very little effect on vaccine efficacy.

We also observed a surprising lack of impact of comorbidities on *Seroconversion*. This result might be because our models considered comorbidities only as present or absent which provided little resolution of specific illnesses or most of the effect of comorbidities was accounted for by the age effect. Indeed, when we explored the contributions of individual comorbidities on *Seroconversion* (Appendix Table S3), we observed some differences in predictability. The results suggested independent effects of specific comorbidities on *Seroconversion*, compared to the effects of other comorbidities and participants without comorbidities. The most frequent comorbidities were neurological disorders in younger participants, while they were related to metabolism in older participants. We found that depression had a significant effect on *Seroconversion* in *Adult1:* adults diagnosed with depression had a lower seroconversion than those without any comorbidities. Similarly, high cholesterol levels had a significant effect on *Seroconversion* in *Adult2*. This result is consistent with the findings above: while high BMI had no predictive value for *Seroconversion* in the *Adult2* subpopulation, accounting for high cholesterol levels improved the modeling outcomes (Appendix Table S3).

While these results are intriguing, depression and high cholesterol accounted for only small fractions of comorbidity occurrences (3 and 10% of *Adult1* and *Adult2* subpopulations, respectively; Appendix Table S3). While these small numbers forced us to use a simplification in our model, that is, accounting for comorbidities only as present/absent, future cohort studies might focus on participants with specific health backgrounds to explore these intriguing relationships further.

## Discussion

We systematically examined the effects of nine factors on *Seroconversion* in human recipients of the flu vaccine, that is, the response to the vaccine 3–4 weeks post‐vaccination, and *BaselineSY*, that is, the HAI titer levels in the subsequent year. To do so, we used a large cohort study with > 1,300 vaccination events (Fig 1). We predicted *Seroconversion* and *BaselineSY* across three subpopulations based on participant age with an overall high accuracy: Categorical classifications were correct in > 3/4 of the cases and numerical values for > 1/3 of the cases across most subpopulations (Fig 5A and B). The remaining, unexplained variation is due to additional factors or non‐linear effects not considered in the model—and forms the basis for future investigation.

Further, we evaluated the contributions of the factors to the prediction of *Seroconversion and BaselineSY* (Figs 6 and 7). Across all age groups, the most predictive factors were prior vaccination status and baseline titer level, followed by age and month of vaccination. In adults, prior vaccination and baseline titer level explained the majority of *Seroconversion*; the effect was smaller in *Children* perhaps due to a still maturing immune system. Further, the effect of prior vaccinations was detectable for at least three years post‐vaccination (Appendix Fig S1), indicating that antibody longevity is essential to consider when evaluating the vaccine response. These findings underscore the importance of pre‐existing immunity when evaluating vaccine efficacy, that is, high baseline HAI titer levels against respective strains and longevity of the antibodies formed during prior infections or vaccinations.

These results are consistent with recent findings (Boyd & Jackson, 2015; Boyd & Jackson, 2015; HIPC‐CHI Signatures Project Team & HIPC‐I Consortium, 2017; Gonzalez‐Dias *et al*, 2020; Kotliarov *et al*, 2020; Tsang *et al*, 2020)—the unique contribution of our work is the simultaneous and predictive modeling to evaluate all nine factors in a unified model. As a result, we showed that in all three subpopulations, prior vaccination and baseline titer level had independent contributions to *Seroconversion*, that is, prior vaccination status and baseline titer level were not fully interchangeable when making predictions (Figs 3 and 6).

Our work compares well with similar approaches. For example, after correcting for baseline titer levels, a new score called adjMFC successfully identified new markers of vaccine efficacy in a transcriptomic dataset (HIPC‐CHI Signatures Project Team & HIPC‐I Consortium, 2017; Tsang *et al*, 2020). However, in contrast to adjMFC, our models included not only baseline titer levels but a total of nine confounding variables, providing a highly comprehensive analysis of a large cohort study that robustly controlled for confounding effects and distentagled the specific, independent effect sizes of the factors.

Our results are validated by previous findings (Dataset EV2). For example, two studies on smaller cohorts confirmed the negative effect of prior vaccination on *Seroconversion* (Gouma *et al*, 2020; Sung *et al*, 2021). Further, a cross‐cohort analysis confirmed the negative effect of age on *Seroconversion* and seroprotection, that is, the actual titer levels (Goodwin *et al*, 2006). While this analysis used a large dataset, it lost accuracy in the estimation of the effect size of age, as several continuous variables had been treated as categorical. Further, our models showed that participant sex affects *Seroconversion* in the *Adult 2* subpopulation, which is consistent with previous work (Klein & Flanagan, 2016; Fink *et al*, 2018; Voigt *et al*, 2019). In contrast to a previous study (DiazGranados *et al*, 2013), we did not find a positive effect of the high‐dose vaccine in the *Adult2* subpopulation (Fig 6). Consistent with a recent finding examining the effect of time of vaccination in a flu season (September to February)(Penkert *et al*, 2021), we found that *Seroconversion* in *Children* and *Adult1* subpopulations was higher when the vaccine was administered later. As most participants in the *Adult2* subpopulation received the vaccine in September or October of a season, we could not evaluate the effect of vaccination time in this group. While the consistency with other findings increases confidence in the validity of our results, the advance in our work lies in simultaneous modeling of multiple factors, rather than examination of a single factor on its own as has been done previously.

Surprisingly, we found that age was not a predictor of the vaccine response amongst adults older than 65 years (Fig 6), seemingly conflicting with recent findings on increased flu severity among the elderly due to immunosenescence (Gounder & Boon, 2019; Huang *et al*, 2019). However, this result is not a contradiction but an extension of these previous findings: we showed that, indeed, participants older than 65 years have lower *Seroconversion* compared to younger participants, but *within* this group, increased age does not have any additional negative effect. The result suggests that perhaps, beyond a specific age, immunosenescence reaches “saturation”.

Our results also contribute to the discussion on the role of obesity in infection and vaccination. While obesity is a strong predictor of flu severity (Honce & Schultz‐Cherry, 2019; Honce *et al*, 2020), conflicting findings exist on its effect on the response to vaccination. In response to an inactivated trivalent influenza vaccine, obese individuals have higher seroconversion than normal weight individuals, but their antibodies are shorter lived (Sheridan *et al*, 2012). In contrast, other studies found obesity to impact seroconversion negatively in both young and old individuals *(*Frasca *et al*, 2016 *)*, or to have no influence on the response in the elderly or in children (Talbot *et al*, 2012; Callahan *et al*, 2014). We find that BMI has no or minor contributions to the vaccine response across the age groups (Figs 6 and 7). We hypothesize that these inconsistencies arose from possible shortcomings in using BMI as a measure of metabolic health (Nuttall, 2015), as well as the mixed effect of confounding factors, primarily age.

Our results illustrate the complexity of the effects of multiple factors on the response to vaccination, their non‐linear effects and interactions, which continues to render prediction of vaccine efficacy a challenge. While our work presents a comprehensive analysis of the factors affecting seroconversion, many additional variables exist that might affect the outcome. For example, genetic makeup, pregnancy, and even the microbiome have been shown to affect severity of a flu infection (Ghedin & Schultz‐Cherry, 2017; Kenney *et al*, 2017; Borges *et al*, 2018) and might have to be considered in future cohort studies.

While it was not the primary goal of our analysis, our findings have potential implications of clinical relevance. For example, our results indicate that the use of the high‐dose vaccine in the older population has little effect, but a “booster shot” later in the season might promote higher overall and more sustained titer levels in the vulnerable subpopulation. The same applies to younger individuals with certain comorbidities, for example, depression or high cholesterol. However, none of these conclusions should be considered as recommendations prior to further evaluation, for example with respect to the confounding impact of concurrent influenza infections on the effect of vaccination month.

The results of our study also present an opportunity to help the identification of novel molecular markers of the vaccine response. For example, one could derive a new score, that is, a *Corrected Seroconversion* score, as the residual of the observed and predicted *Seroconversion*, which simultaneously accounts for a number of known factors that typically confound the response to vaccination, such as vaccination history, age, BMI, sex, and month of vaccination. Using the *Corrected Seroconversion* would allow identifying molecular markers of the vaccine response *independent* of what can be explained by the participant’s specific background, in a highly quantitative manner. Such score could, therefore, complement the traditional screening which employs uncorrected *Seroconversion* or other measures.

## Materials and Methods

### Data pre‐processing

As part of an ongoing study by the University of Georgia, Athens (UGA), a total of 690 participants had been recruited during five seasons between 2016 and 2020 (UGA1‐5). The study procedures, informed consent, and data collection documents were reviewed and approved by the Institutional Review Board of the University of Georgia (IRB #3773)). Participants received the split‐inactivated influenza vaccine Fluzone by Sanofi Pasteur. For the 2016–2017 season, influenza strains included in the vaccine formulation were as follows: A/California/09 (H1N1), A/Hong Kong/2014 (H3N2), B/Phuket/2013 (Yamagata lineage), and B/Brisbane/2008 (Victoria lineage). For the 2017–2018 season, the strains were A/Michigan/15 (H1N1), A/Hong Kong/2014 (H3N2), B/Phuket/2013 (Yamagata lineage), and B/Brisbane/2008 (Victoria lineage). For the 2018–2019 season, the strains were A/Michigan/15 (H1N1), A/Singapore/16 (H3N2), B/Phuket/2013 (Yamagata lineage), and B/Colorado/2017 (Victoria lineage). For the 2019–2020 season, the strains were A/Brisbane/2018 (H1N1), A/Kansas/2017 (H3N2), B/Phuket/2013 (Yamagata lineage), and B/Colorado/2017 (Victoria lineage). For the 2020–2021 season, the strains were A/Guangdong‐Maonan/2019 (H1N1), A/Hong Kong/2019 (H3N2), B/Phuket/2013 (Yamagata lineage), and B/Washington/2019 (Victoria lineage). The strains are briefly listed in Fig 1A. Adults older than 65 years were offered the High‐Dose (HD) option which contained four‐fold the amount of each vaccine strain compared to Standard‐Dose (SD). For the first four seasons (2016–2019), Yamagata lineage was not included in HD. For the fifth season (2020), all four strains were included in HD. All vaccines were administered intramuscularly from prefilled single‐dose syringes without preservative or adjuvant. The dose and HA amounts were as follows: UGA1–4: SD—Quadrivalent, 0.5 ml dose with 60 μg HA (15 μg/strain); HD—Trivalent, 0.5 ml dose with 180 μg HA (60 μg/strain); UGA5: SD—Quadrivalent, 0.5 ml dose with 60 μg HA (15 μg/strain); HD—Quadrivalent, 0.7 ml dose with 240 μg HA (60 μg/strain).

Participants provided blood samples on the day of vaccination (day 0) (sample collected before the vaccination event) and 21/28 days post‐vaccination (day 21/28). Hemagglutination inhibition assays were performed on the blood samples against each of the vaccine strains as well as other strains. Demographic data including age, body mass index (BMI), sex, race, comorbidities, prior vaccination status, as well as month of vaccination in a flu season, vaccine dose, and baseline (D0) HAI titer levels or post‐vaccination D21/28 titer levels were used for *Seroconversion* and *BaselineSY* predictions.

We used *Seroconversion* as log_2_‐transformed ratio of HAI titer levels at day 21/28 (D21/28) and day 0 (D0) for each strain, and a composite *Seroconversion* as the sum of log_2_‐transformed ratios across the strains, as proposed in a previous study (Abreu *et al*, 2020). We tested whether the HAI titer levels used here present a valid measure of protection against subsequent flu infections. For a very small number of participants (*n* = 17) in the UGA4 andUGA5 cohorts, we had information on presence or absence of influenza A infection (3 and 14, respectively) in the 2020–2021 season (UGA5) after receiving the vaccine in the year prior (UGA4) (Appendix Table S4). The D28 HAI titer levels against A/H1N1 in the UGA4 season for the 14 influenza A negative participants were significantly higher compared with those 3 positive participants (*P*‐value = 0.002; Appendix Table S4), indicating that high titer levels provide protection against flu infections.

Further, we log_2_‐transformed age and BMI to account for their potential non‐linear impact. To predict *Seroconversion*, we used the log_2_‐transformed baseline D0 HAI titer levels, age, BMI, as well as the other demographic factors (variables). To predict *BaselineSY*, we used the same variables, except that the log_2_‐transformed D0 HAI titer is replaced with log_2_‐transformed D21/28 HAI titer (Fig 4). We assembled Baseline cohorts consisting of 672 vaccination events comprising data for participants for whom the study comprised data in two continuous seasons, that is, between UGA1 and 2, 2 and 3, 3 and 4, and 4 and 5, respectively. The Baseline cohorts were used for *BaselineSY* prediction.

We performed predictions in three subpopulations separately: *Children* (< 18 years), *Adult1* (18–64 years), and *Adult2* (≥ 65 years). The subpopulations were defined based on what is common in this field and on the fact that the high‐dose version of the vaccine was offered as an option only to adults ≥ 65 years—so the effect of dose can only be modeled among *Adult2* participants.

### Predictive modeling and evaluation with an independent cohort

We first split each dataset into training and test data sets. To ensure independent evaluation of the predictions, the test sets were not used to construct the models. For *Seroconversion* prediction, we used UGA cohorts 1–3 and 5 as the training set and cohort 4 as the test set. Similarly, we used Baseline cohorts 1–2 and 4 as the training set and cohort 3 as the test set. The decision was made based on the overall composition of the cohorts. UGA1‐3 were biased toward recruitment of specific subpopulations. UGA5 was biased toward returning participants and potentially skewed to the overlap with the SARS‐CoV2 pandemic.

We then tested different machine learning methods in WEKA environment (Ivanciuc, 2008) (https://sourceforge.net/projects/weka/), including Random Forest and Neural Network, and selected multiple linear regression (MLR) as it had the best performance. So we applied MLR to model the effect of each variable and to predict *Seroconversion* and *BaselineSY*, for individual strains as well as the composite score. To reduce redundant variables and avoid overfitting, we first performed feature selection on the training set. We used WrapperSubsetEval attribute evaluator with the internal classifier set to be LinearRegression, and BestFirst search method, as well as a 10‐fold cross‐validation setting to do feature selection. Variables that were selected ≥ 1 time out of 10 times were retained. We then obtained the optimal MLR model with the selected variables with the training set, with default settings. Finally, we evaluated the performance of the model with the test set, by both *R* ^2^ and the accuracy of predicting categories (Fig 4).

The strain‐specific modeling followed the same procedures as the modeling for the composite scores, but was performed for the four vaccine strains separately. This modeling did not account for changes in the actual vaccine strains used in each season (Fig 1A). As Appendix Fig S7 shows, this assumption is valid due to cross‐reactivity between antibodies built against different strains of the same subtype: HAI titer levels for both the A/H1N1 and A/H3N2 strains used in the UGA4 cohort correlate well with the respective titer levels against the A/H1N1 and A/H3N2 strains used in the UGA1‐3 cohorts.

### Estimating the relative importance of variables

We estimated the relative importance of each variable with the Leave‐One‐Covariate‐Out (LOCO) method as described previously (Lei *et al*, 2018). Briefly, we removed one variable out at a time from the MLR model, obtained a new *R* ^2^, calculated the decrease in *R* ^2^ as a percentage defined as (*R* ^2^_full model_ − *R* ^2^_reduced model_)/*R* ^2^_full model_), and used this metric as the relative importance of the variable. Appendix Tables S2 and S3 show the results of this evaluation.

### Power analysis

To evaluate the optimal sample size for this study, we performed statistical power and sample size analysis using standard parameters and various effect sizes. We used the function pwr.f2.test() in R package pwr which calculates the power given the following input: sample size, number of predictors, effect size, and significance level. Accounting for sample size in the respective subpopulations, the number of predictors in the individual models, and effect size of the models, we estimated to have ~ 100, ~ 100, and 97% power in the *Seroconversion* prediction results, and 99.99999, ~ 100, and ~ 100% power in the *BaselineSY* prediction results, for three subpopulations *Children*, *Adult1*, and *Adult2*, respectively (Appendix Table S5).

## Author contributions

**Shaohuan Wu:** Conceptualization; Data curation; Software; Formal analysis; Validation; Investigation; Visualization; Methodology; Writing—original draft; Writing—review & editing. **Ted M Ross:** Resources. **Michael A Carlock:** Resources. **Elodie Ghedin:** Supervision; Funding acquisition; Writing—review & editing. **Hyungwon Choi:** Data curation; Formal analysis; Supervision; Methodology; Writing—review & editing. **Christine Vogel:** Conceptualization; Formal analysis; Supervision; Funding acquisition; Investigation; Visualization; Methodology; Writing—original draft; Project administration; Writing—review & editing.

In addition to the CRediT author contributions listed above, the contributions in detail are:

CV and SW conceptualized this project; TD and MC conducted the flu vaccine cohort study and provided the data; SW performed the data analysis; SW and CV wrote the manuscript; EG and HC edited the manuscript; HC provided valuable computational advice throughout the analysis.

## Disclosure and competing interests statement

The authors declare that they have no conflict of interest.

## Supporting information



Appendix

Dataset EV1

Dataset EV2

## Data Availability

All scripts and models were deposited on github (https://github.com/sw5019/Fluvacc‐metadata‐project).
