# Supplementary material for: Evaluation of determinants of the serological response to the quadrivalent split‐inactivated influenza vaccine
Source: Mol Syst Biol. 2022 May 6;18(5):e10724. doi: 10.15252/msb.202110724 (PMC9073386; doi:10.15252/msb.202110724)
Supplement: Supplementary file 1 — Appendix [file MSB-18-e10724-s003.pdf]

Appendix

Evaluation of determinants of the serological response to the quadrivalent split-inactivated influenza vaccine

Shaohuan Wu[1], Ted M. Ross[2,3], Michael A. Carlock[2,3], Elodie Ghedin[1,4], Hyungwon Choi[5], Christine Vogel[1*]

[1] Center for Genomics and Systems Biology, New York University, New York, USA;

[2] College of Veterinary Medicine, Department of Infectious Diseases, University of Georgia, Athens, Georgia, USA;

[3] Center for Vaccines and Immunology, University of Georgia, Athens, Georgia, USA;

[4] Systems Genomics Section, Laboratory of Parasitic Diseases, NIAID, NIH, Bethesda, MD, USA;

[5] Department of Medicine, Yong Loo Lin School of Medicine, National University of Singapore, Singapore,

Table of contents

Figure S1. Impact of prevaccination history on seroconversion **2**

Figure S2. Intercorrelations between all priors. **3**

Figure S3. Observed vs. predicted values for Seroconversion and BaselineSY, respectively **4**

Figure S4. Predicting Seroconversion without using the baseline HAI titer levels. **5**

Figure S5. Predicting Seroconversion for people that only participated once in the UGA study. **6**

Figure S6. Predicting Seroconversion for events arising from first-time and second-time participation for participants who participated twice or more in the UGA study **7**

Figure S7. Cross-reactivity of antibodies built against different vaccine strains from the same subtype. **8**

Figure S8. Predicting Seroconversion in Adult1 with data that fall within the first three months of the five flu seasons **9**

Table Legends **10**

Table S1 **13**

Table S2 **14**

Table S3 **15**

Table S4 **16**

Table S5 **17**

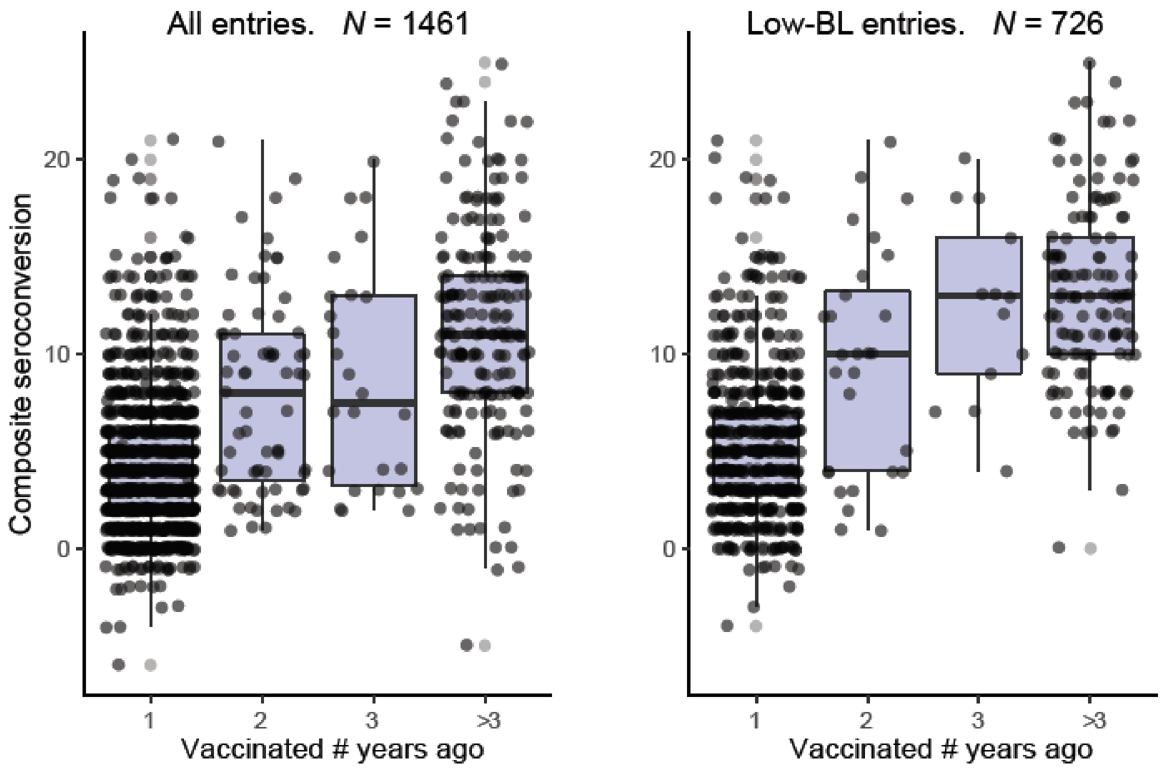

Figure S1. Impact of prevaccination history on seroconversion

Left panel: all participants (*N* = 1,461); Right panel: participants with a low baseline (BL; seropositive strains =<2; *N* = 726).

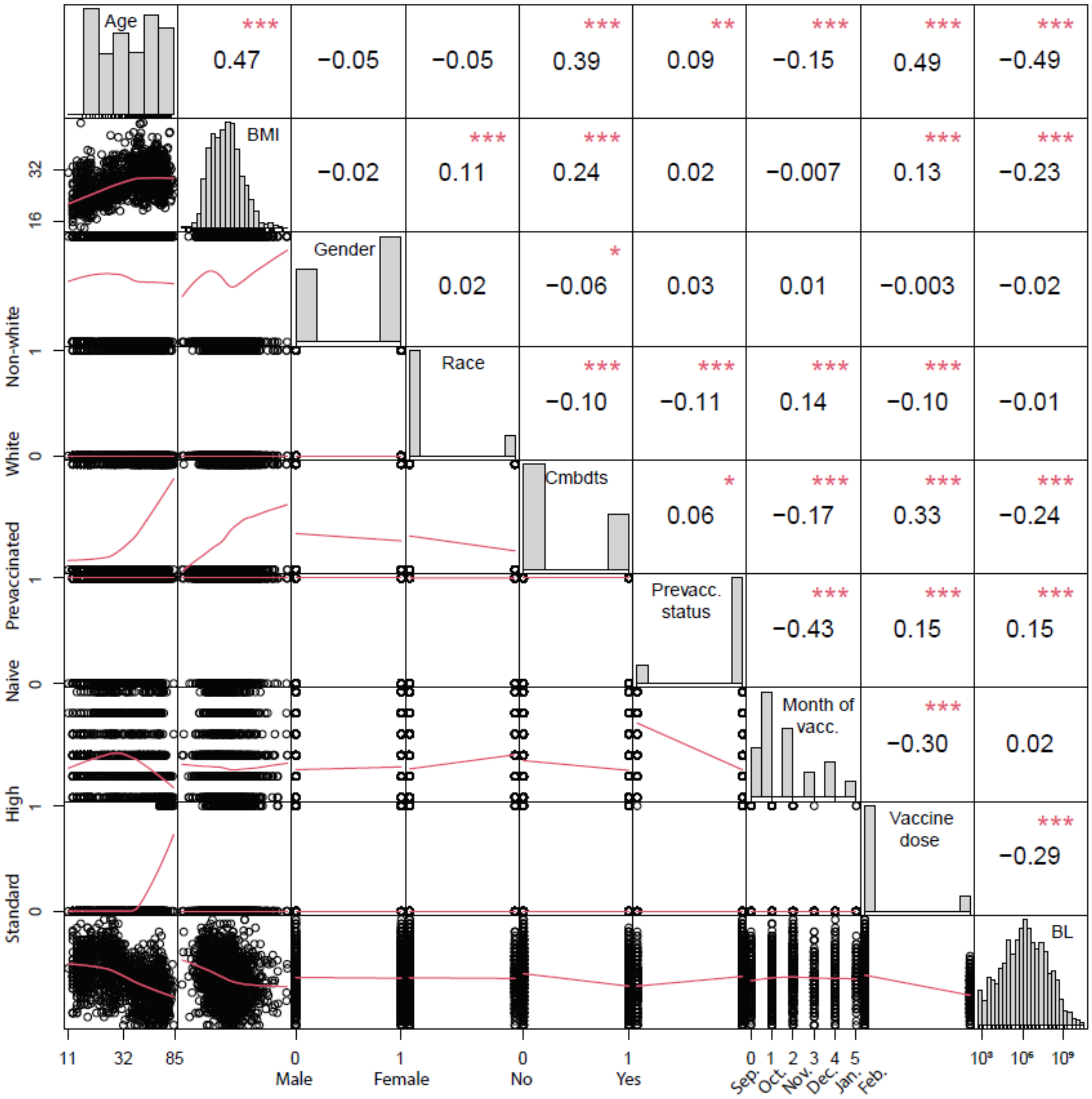

Figure S2. Intercorrelations between all priors.

The table shows correlations between all variables that are included in the modeling. Age and BMI are log2-transformed, and the categorical variables are transformed into numeric values first, then Pearson's correlation is calculated between the variables. *, P<0.05; **, P<0.01; ***, P<0.001. Plots along the diagonal are distributions of the variables. The red lines in the correlation plots below the diagonal are loess regression lines. Cmbdts: comorbidities; Prevacc: prevaccination; Vacc: vaccination; BL: baseline - here composite baseline.

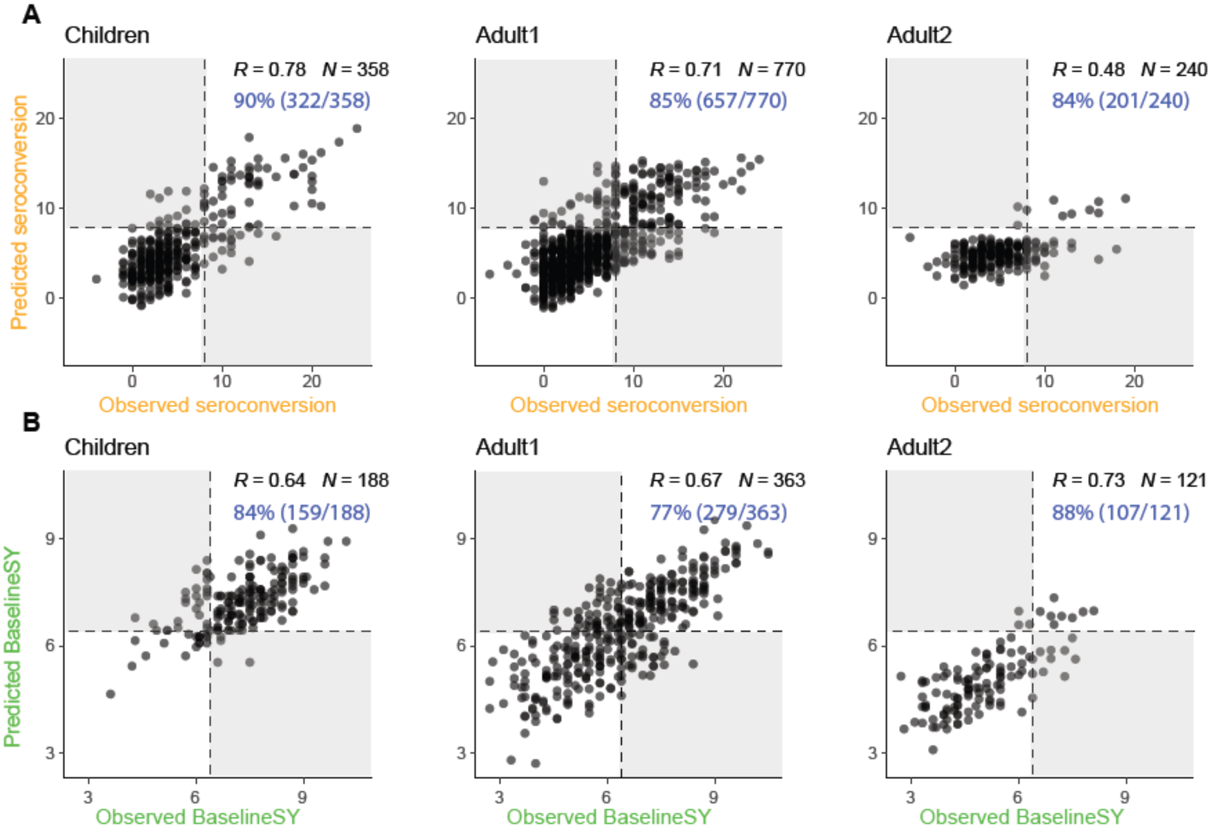

Figure S3. Observed vs. predicted values for *Seroconversion* and *BaselineSY*, respectively

Similar to Figure 4, only with all data points plotted. The conclusions are identical to those described in the main text.

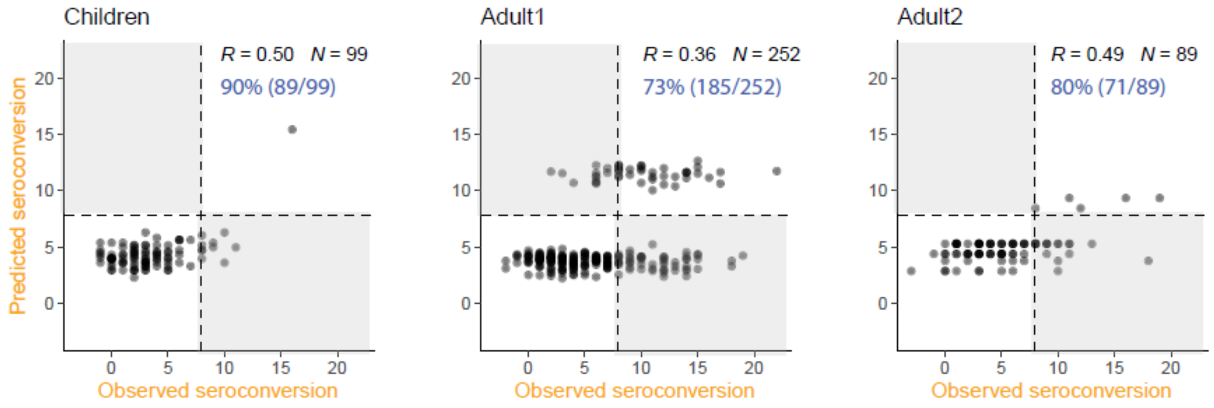

Figure S4. Predicting *Seroconversion* without using the baseline HAI titer levels.

Similar to Figure 4, but the model was built without the prior on the baseline HAI titer.

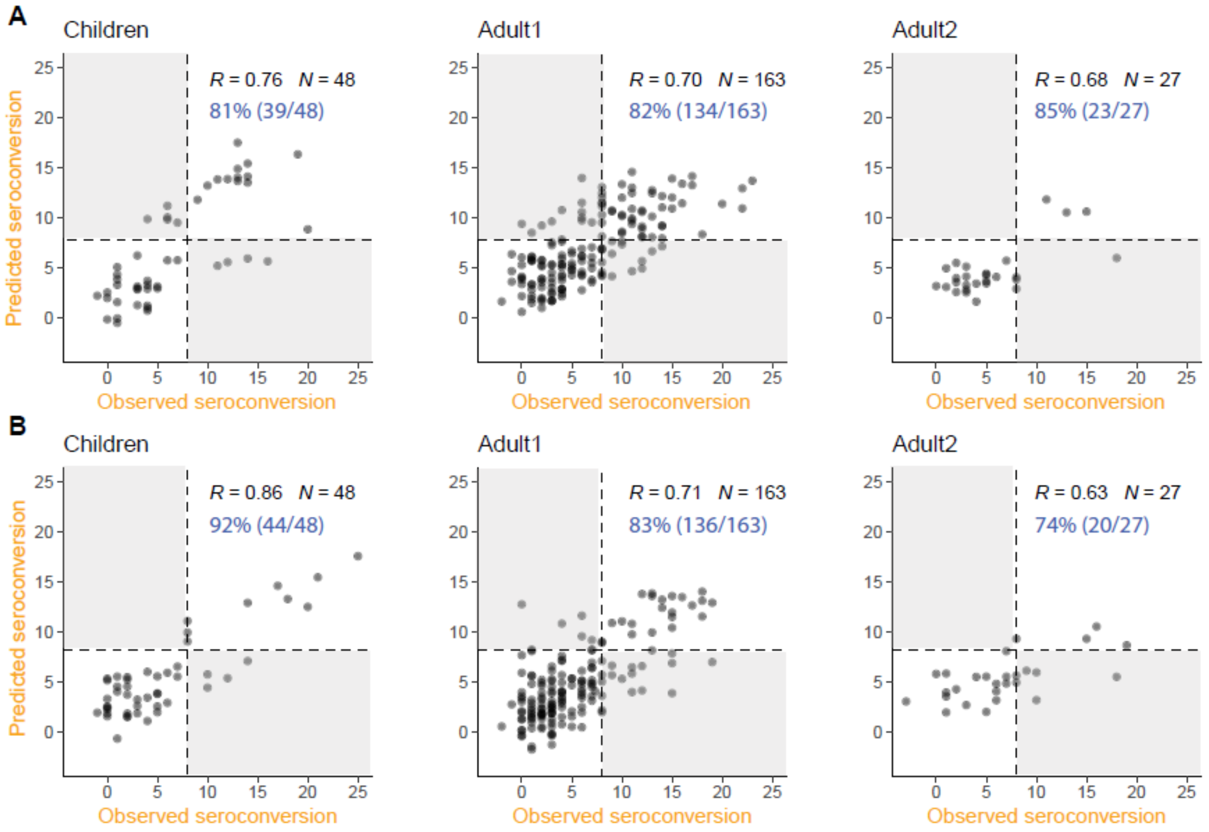

Figure S5. Predicting Seroconversion for people that only participated once in the UGA study.

A. Observed and predicted seroconversion for one-time participants in three subpopulations. B. Observed and predicted seroconversion for the same number of randomly selected vaccination events in three subpopulations.

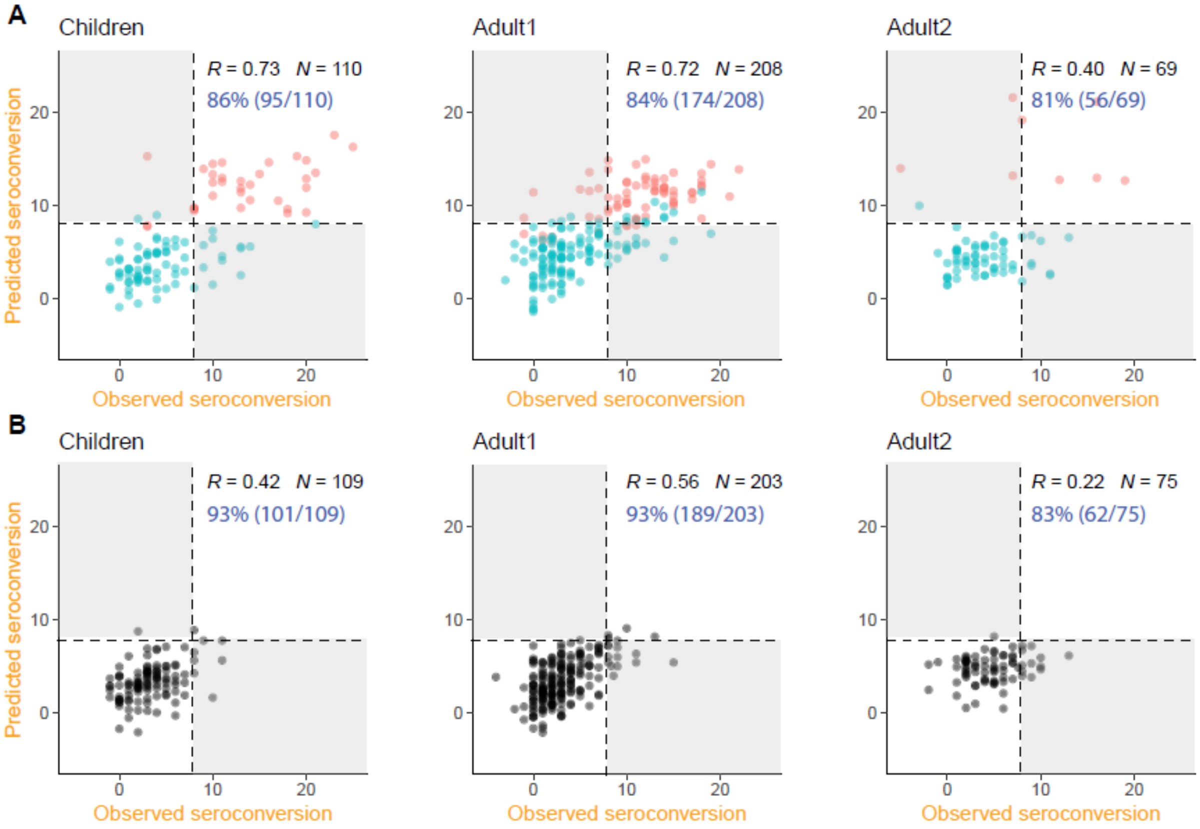

Figure S6. Predicting *Seroconversion* for events from first-time and second-time participation for participants who participated twice or more in the UGA study

For the same participants who participated twice or more in the UGA study. A. Observed and predicted seroconversion in response to the first vaccination. Red dots depict ‘naive’ participants, i.e. those without vaccination in the three years prior to the vaccination event shown here. Cyan dots depict ‘pre-vaccinated’ participants, i.e. those with vaccination in the year prior to the vaccination event shown here B. Observed and predicted seroconversion in response to vaccination in the subsequent year. All participants, by definition, are prevaccinated, as they received the vaccine through the UGA study in the year before.

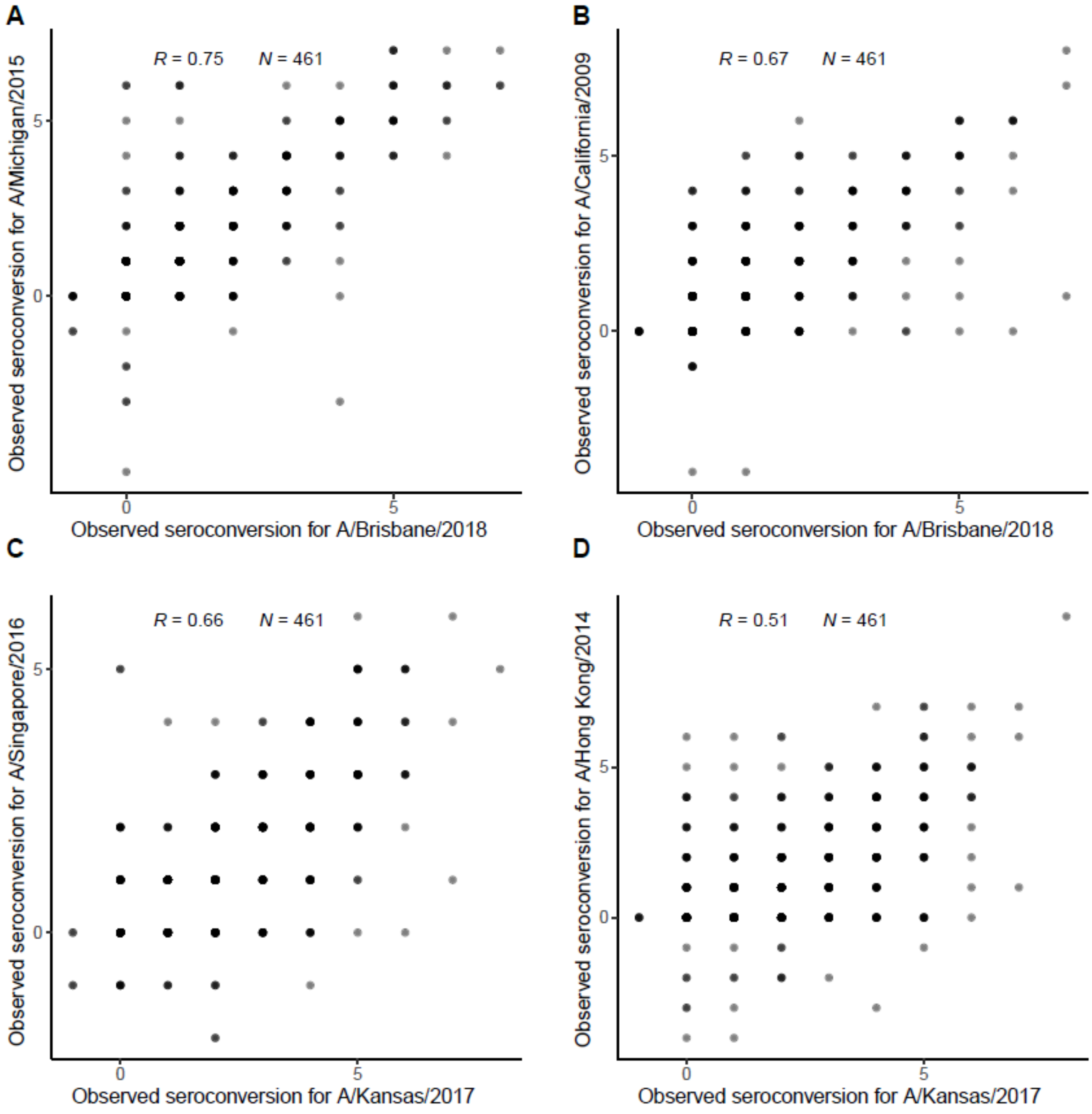

Figure S7. Cross-reactivity of antibodies built against different vaccine strains from the same subtype.

While the quadrivalent vaccine included only one strain of each subtype, HAI titers at D0 and D28 were measured against a number of other strains. Correlation between observed seroconversion in one strain with observed seroconversion in another strain suggests that, regardless of the strain used in the vaccine, antibodies cross-react with other strains. The figure shows the correlation of seroconversion observed in the UGA4 cohort against the vaccine strain (**A, B.** A/H1N1: Brisbane/2/2018; **C, D.** A/H3N2: Kansas/14/2017) and other strains of the same subtype used in UGA1 to UGA3, as specified in **Figure 1** (see main text).

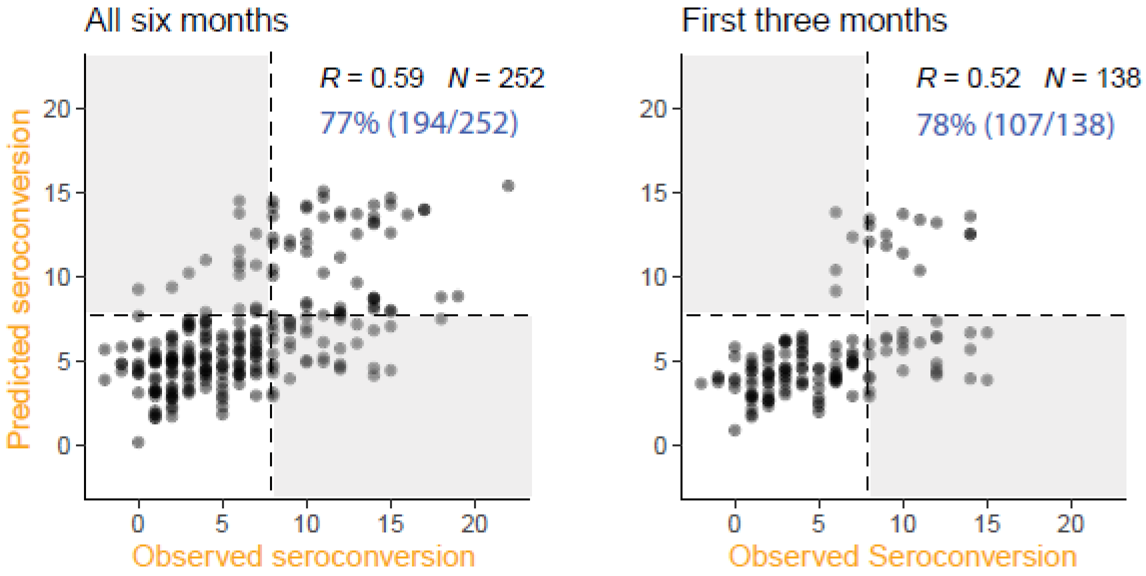

Figure S8. Predicting *Seroconversion* in *Adult1* with data that fall within the first three months of the five flu seasons

Left panel: prediction result with data entries from all six months; Right panel: prediction result with data entries that fall within the first three months.

Appendix Table Legends

Table S1. Contributions of individual priors for *Seroconversion* and *BaselineSY* prediction, respectively.

The table shows the data underlying **Figure 6**.

Table S2. Contributions of individual priors for *Seroconversion* and *BaselineSY* prediction, respectively, for individual strains.

The table shows the data underlying **Figure 7**.

Table S3. Distributions of the top abundant comorbidities in three subpopulations and their effects on seroconversion.

The table shows the frequencies of the top three frequent comorbidities in three subpopulations and their effects on seroconversion. The first column is subpopulation, second column the top three frequent comorbidities, third column the number of data entries for participants with this comorbidity, fourth column the total number of vaccination events, and fifth column the frequency of each comorbidity in each subpopulation. The sixth column is the comparison of “corrected seroconversion” (or residual seroconversion), i.e., observed minus predicted seroconversion, which represents the serological response after controlling for all the other factors, between participants with each of the listed comorbidities and those without any comorbidities, and seventh column the comparison of
“corrected seroconversion” between participants with each of the listed comorbidities and those with other comorbidities other than the listed three, in each subpopulation.

Table S4. D28 HAI titer levels of participants in UGA4 who had the influenza infection information recorded in UGA5

We tested the difference in D28 HAI titer levels against A/H1N1 or A/H3N2 between 3 A positive and 14 negative participants and the p-values are 0.002 and 0.35 respectively. We also tested the difference in D28 HAI titer levels against B/Yam or B/Vic between 2 B positive and 14 negative participants and the p-values are 0.53 and 0.27 respectively. In summary, a significant difference is detected between the D28 HAI titer levels against A/H1N1 between flu A positive and negative participants.

Table S5. Parameters used to calculate the power of the modeling results for *Seroconversion* and *BaselineSY* predictions in three subpopulations

Seroconversion and *BaselineSY* are the composite metrics here. For each subpopulation, we used the number of unique participants in the training set as the sample size, so the power value we obtained is conserved because we used vaccination events in the prediction. The predictors are those retained in each model shown in **Table S1**. Effect size here is defined as $R^2/(1-R^2)$, and we set the significance level to be 0.05.

Appendix Table S1

Relative contribution for SC prediction				Relative contribution for BL prediction			
Variable	Children	Adult1	Adult2	Variable	Children	Adult1	Adult2
Age	0.13	0.11	NA	Age	NA	-0.03	NA
BMI	NA	0.03	NA	BMI	NA	NA	NA
Gender	NA	NA	0.16	Gender	0	NA	0
Race	NA	NA	NA	Race	NA	NA	NA
Comorbidities	NA	0	NA	Comorbidities	NA	NA	0
PreVacc. status	0.23	0.09	0.84	PreVacc. status	0.08	NA	NA
Month of vacc.	0.05	0.09	NA	Month of vacc.	NA	0.03	-0.21
Vaccine dose	NA	NA	-0.16	Vaccine dose	NA	NA	-0.1
D0 HAI titer	0.38	0.63	0.04	D28 HAI titer	0.97	0.78	1

Appendix Table S2

	Relative conribution for SC prediction											
	H1N1			H3N2			IBV_Yam			IBV_Vic		
Variable	Children	Adult1	Adult2	Children	Adult1	Adult2	Children	Adult1	Adult2	Children	Adult1	Adult2
Age	0.05	0.13	-0.5	NA	NA	NA	NA	0.04	NA	0.05	NA	NA
BMI	NA	NA	NA	-0.11	NA	0.4	NA	NA	NA	NA	NA	NA
Gender	NA	NA	NA	NA	NA	NA	NA	NA	NA	NA	NA	0
Race	NA	NA	NA	0.04	0	NA	-0.03	NA	NA	NA	0	NA
Comorbidities	NA	NA	NA	NA	NA	NA	NA	NA	NA	NA	NA	NA
PreVacc. status	0.05	-0.13	0.83	0.11	0.17	0.5	0.12	0.21	0.61	0.07	0.14	0.34
Month of vacc.	0	NA	NA	0	NA	NA	-0.03	0.04	-0.13	-0.03	0	NA
Vaccine dose	NA	NA	-0.17	NA	NA	0	NA	NA	NA	NA	NA	NA
D0 HAI titer	0.95	0.83	0.67	0.59	0.83	0.2	0.79	0.58	0.65	0.66	0.72	0.34
	Relative conribution for BL prediction											
	H1N1			H3N2			IBV_Yam			IBV_Vic		
Variable	Children	Adult1	Adult2	Children	Adult1	Adult2	Children	Adult1	Adult2	Children	Adult1	Adult2
Age	-0.07	0.01	-0.11	-0.25	-0.2	NA	NA	NA	NA	NA	NA	NA
BMI	-0.13	NA	NA	-0.38	NA	NA	NA	NA	NA	NA	NA	NA
Gender	NA	NA	0	0.13	NA	0.17	NA	NA	NA	-0.15	NA	-0.13
Race	NA	NA	NA	NA	NA	NA	0	0	NA	NA	NA	NA
Comorbidities	NA	NA	0	NA	NA	NA	NA	NA	NA	0	0.04	NA
PreVacc. status	-0.09	NA	NA	NA	NA	NA	NA	NA	NA	0.26	0.03	NA
Month of vacc.	NA	NA	0.08	0	0	-0.08	NA	0	-0.03	NA	0.03	-0.29
Vaccine dose	NA	NA	NA	NA	NA	NA	NA	NA	0	NA	NA	-0.42
D28 HAI titer	0.98	0.83	0.98	1	0.85	1	0.46	0.9	1	1	1	1

Appendix Table S3

Subpopulation	Top three comorbidities	#	# total	Fraction	SC* comparison - with those without any comorbidities	SC* comparison - with those with other comorbidities other than the listed three
Children	Asthma	12	359	0.03	0.60	0.32
	Anxiety	9	359	0.03	0.32	0.19
	Adhd	7	359	0.02	0.12	0.07
Adult1	Hypertension	65	769	0.08	0.64	0.64
	Depression	23	769	0.03	0.03 *	0.16
	Diabetes	15	769	0.02	0.65	0.90
Adult2	Hypertension	59	240	0.25	0.74	0.44
	High Cholesterol	24	240	0.10	0.02 *	0.07
	Hypothyroidism	19	240	0.08	0.46	0.12

Appendix Table S4

	From UGA4				From UGA5
Participant ID	D28_Titer_A/H1N1	D28_Titer_A/H3N2	D28_Titer_B/Yam	D28_Titer_B/Vic	Flu test result
A367	10	5	80	10	A Positive
A384	5	80	320	10	A Positive
A387	10	10	160	80	A Positive
A34	160	160	640	160	B Positive
A373	10	20	80	320	B Positive
A83	10	5	20	20	Negative
A118	80	320	80	160	Negative
A170	20	80	20	10	Negative
A190	40	20	320	160	Negative
A196	5	20	20	5	Negative
A221	80	160	160	80	Negative
A225	40	10	320	20	Negative
A273	10	5	20	40	Negative
A275	40	20	320	80	Negative
A307	20	20	20	80	Negative
A321	10	20	40	20	Negative
A424	40	5	40	80	Negative
A426	40	160	80	80	Negative
A494	80	80	40	160	Negative

Appendix Table S5

Prediction goal	Subpopulation	Sample size	# predictors	Effect size
Seroconversion	*Children*	156	4	1.69
	Adult1	312	6	1.35
	Adult2	102	4	0.21
BaselineSY	*Children*	102	3	0.62
	Adult1	255	3	1.78
	Adult2	72	5	1.55
