# Supplementary material for: Evaluation of determinants of the serological response to the quadrivalent split‐inactivated influenza vaccine
Source: Mol Syst Biol. 2022 May 6;18(5):e10724. doi: 10.15252/msb.202110724 (PMC9073386; doi:10.15252/msb.202110724)
Supplement: Supplementary file 4 [file MSB-18-e10724-s001.pdf]

molecular systems biology

Evaluation of determinants of the serological response to the split-inactivated influenza vaccine

Shaohuan Wu, Ted Ross, Michael Carlock, Elodie Ghedin, Hyungwon Choi, and Christine Vogel
DOI: 10.15252/msb.202110724

Corresponding author(s): Christine Vogel (cvogel@nyu.edu) , Shaohuan Wu (sw5019@nyu.edu)

Review Timeline:

Submission Date:	30th Sep 21
Editorial Decision:	16th Nov 21
Revision Received:	17th Mar 22
Editorial Decision:	5th Apr 22
Revision Received:	11th Apr 22
Accepted:	12th Apr 22

Editor: Jingyi Hou

Transaction Report:

(Note: With the exception of the correction of typographical or spelling errors that could be a source of ambiguity, letters and reports are not edited. Depending on transfer agreements, referee reports obtained elsewhere may or may not be included in this compilation. Referee reports are anonymous unless the Referee chooses to sign their reports.)

Thank you for submitting your work to Molecular Systems Biology. We have now heard back from the three reviewers who agreed to evaluate your manuscript. As you will see from the reports below, the reviewers acknowledge the potential interest of the study. They raise, however, a series of concerns, which we would ask you to address in a major revision.

I think that the reviewers' recommendations are rather clear and there is no need to reiterate their comments. Some of the key issues that would need to be addressed are the following:

- In light of Reviewer #1's comments, efforts should be made to clearly highlight the novelty of this study.
- Reviewer #1 raised significant concerns about data analyses, the validation, the cohort size and demography, which need to be carefully addressed and/or discussed.

Other issues raised by the reviewers need to be satisfactorily addressed as well. As you may already know, our editorial policy allows in principle a single round of major revision, and acceptance or rejection of the manuscript will depend on another round of review, it is therefore essential to provide responses to the reviewers' comments that are as complete as possible.

On a more editorial level, we would ask you to address the following issues:

Reviewer #1:

Wu and colleagues aim to identify determinants of vaccine efficacy by modeling of nine variables interrogating an available cohort.

This is a well written manuscript on a fundamental important topic. However, there are already publications assessing some of the variables under consideration in this study although here authors combine all of them under the same framework. Nonetheless, one may argue that the main conclusions of the work were already out there. Therefore, this study provides validation more than a conceptual advance and may fall short even of future use of the models.

Major comments.
1-. It is important to clarify how many different individuals were assessed in this study. The term "vaccination events" may be confusing for a non-expert. In other words, the n should refer to different individuals and as such should be indicated clearly. In

addition, authors should clarify that the same individual was not considered twice as per different vaccination campaign. On the other hand, if data is available for the same individual across different vaccination campaigns authors may consider to present these results and whether the same results were obtained.
2-. The fact that nine variables are analyzed questions whether the study has enough power calculation in each of the subgroups. This reviewer remains unconvinced that there is not enough number of subjects in each of the subgroups to draw any meaningful model and conclusions thereof.
3-. The demography of the cohort is sensible in terms of age groups; however the lack of description of the "comorbidities" makes impossible to judge indeed this is a consistent variable across the age groups. Different comorbidities would have different effect on the variable under consideration.
4-. Although this reviewer fully appreciates this is a computer-based work, the modelling and prediction is as good as it helps to explain the reality. Therefore, this work would have required additional work to validate their findings. In other words, a prospective study to test the prediction of vaccine failure based on the modelling. Otherwise, the conclusions remain at the theoretical level.
5-. The study is based on a very specific population that it is very loosely described (refer also to comment 3), making hard to evaluate the consideration of race, for example. For the age variable, the numbers seem low to make any conclusion. In addition, the authors need to include as an important limitation of their study that their conclusions only apply to a restricted cohort and may not be of global interest.

Minor comments.
The readability of the manuscript will improve if the authors will refer not only the figure number but also the panel when describing the results.

Reviewer #2:

Comments for the authors of the EMBO Molecular Systems Biology manuscript number MSB-2021-10724:
The authors of the EMBO Molecular Systems Biology manuscript "Evaluation of determinants of the serological response to the split-inactivated influenza vaccine", present their efforts to use previous information regarding vaccine-induced immunity as a predictor of vaccine efficacy in specific vaccine groups. To develop this model, they have used more than 1,300 vaccine events, and they measured seroconversion and hemagglutination inhibition (HAI) titers one year post-vaccination. The group took multiple factors into consideration during this evaluation, including age, BMI, sex, race, comorbidities, pre-vaccination history, and baseline HAI. Through use of regression models, they show the strongest determinants for seroconversion are prior vaccination, baseline titer, and age. Interestingly, these three criteria had a negative effect on this outcome. The authors only report a gender effect in older populations, a small effect on vaccine month that was difficult to fully evaluate due to the timing of vaccine delivery in some populations (like the Adult 2 population), and an unexpected minimal impact for BMI (which may be due to the association between BMI and age). Overall, this is an interesting study that uses modeling to predict issues in influenza vaccination that need to be understood at both the basic science and the clinical level. Specifically, understanding the impact of predictive data on clinical outcomes, will be important for driving future vaccination campaigns. The data presented here move closer to this understanding, and I have a few comments listed below that I would like the authors to address during the revision process.

Comments:

1. I greatly appreciated the training of the models using previously collected data (4 seasons) and the prediction developed for the 5th season. While the study focuses on seroconversion results, I was wondering if there was any way to include data regarding influenza infection diagnosis in these individuals. While seroconversion may be affected, the clinical impact is worth adding to the outcomes reported.
2. I would like to learn a little more about how the authors plan to implement these findings into widespread application in the clinical setting. Would application of these findings into the clinical setting require every vaccine recipient to have their HAI tested prior to vaccination? Could there be future recommendations to provide a second vaccine in the older population that would be delivered later in the season (Jan/Feb)?

Reviewer #3:

This is an interesting manuscript with work describing the serological response to influenza vaccination but I found the flow of the manuscript difficult to follow. This might be that I am less familiar with the approaches adopted to the analysis of the data and the way the results are presented, but I feel that the quality of the manuscript could be improved with some re-working of how it is to be presented.

One of the problems I had was with what was an observed result and what was predicted in a test set result and how this was compared with the observed result in the test case. It was also not clear to me what were the models used precisely.

I also think that improvements to the figure legends would greatly clarify what is being shown.

Figure 1. I did not know what the Y-axis represented in the bar charts in panel A.
Figure 2. I was not sure what the Y-axis in the composite baseline (log10) represented. The title itself is not appropriate - figure titles are not usually conclusions (with jargon).
Figure 4. The units for predicted and observed seroconversion and BaselineSY should be fully defined.
Figure 5. The shades of color were difficult to see on my printout for this figure and some of the others.

I think the details of the precise vaccines used should be presented as supplementary materials, noting that in some cases the high dose vaccine was given (P8, second paragraph). The composition of each vaccine delivered should be given.

In addition, I did not understand whether when a vaccine recommendation was made, whether the day 0 titre would have been assessed against the previous vaccine as well as the current vaccine that was delivered at day 0.

The choice of year four (UGA 4, 2019-2020) of the study is interesting and the basis for this rather than UGA 5 (2020-2021) needs some discussion. The authors might then point out that UGA 4 2019-2020 would have been vaccinated with a 3C.3a H3N2 virus, the first time since 2015-2016.

The authors might address whether retrospective data contribute to the predictions. Thus in UGA5, does a titre raised against B/Washington/02/2019 in 2020 predict a titre retrospectively against B/Colorado/06/2017 in 2019? Likewise, does a titre raised against B/Brisbane/60/2008 predict a titre raised against in a subsequent year against B/Colorado/06/2017.

It is interesting that the authors suggest for the Adult1 panel that seroconversion and BaselineSY were predicted worst. They also note that this group has the widest range of vaccination dates (Figure 1). Do these correlate? (Perhaps following infection and not vaccination).

P4, paragraph six. I was not sure why it was expected that prior vaccination and high baseline levels did not predict high seroconversion. The authors should elaborate.

P5, paragraph two. The authors note a marked effect on B/Yamagata seroconversion. The authors might ask whether this is associated with the widespread circulation of the B/Yamagata lineage viruses in 2017-2018?

Reviewer #1:

Wu and colleagues aim to identify determinants of vaccine efficacy by modeling of nine variables interrogating an available cohort.

This is a well written manuscript on a fundamental important topic. However, there are already publications assessing some of the variables under consideration in this study although here authors combine all of them under the same framework. Nonetheless, one may argue that the main conclusions of the work were already out there. Therefore, this study provides validation more than a conceptual advance and may fall short even of future use of the models.

> **The revised manuscript contains a new Appendix Table S4 which describes related work as well as an expanded discussion in the Discussion section (p. 8). We argue that prior work has mostly analyzed only one or few variables at a time, but not nine factors simultaneously as we have done. Prior work also often reduced predictive accuracy of the work by using categorical variables rather than the quantitative values, and/or did not thoroughly evaluate the predictions. While similar in concept and goal to existing studies, our study represents the most comprehensive one of its kind.**
>
> **Doing so enabled us to gain new insights that expand previous findings. For example, we disentangled the previously controversial role of BMI on flu vaccine response (p. 8) and defined the relationship between prior vaccination status and baseline titer level (p. 7). We also expanded the discussion of the new insights arising from our work (Discussion, p. 7-9).**

Major comments.

1-. It is important to clarify how many different individuals were assessed in this study. The term "vaccination events" may be confusing for a non-expert. In other words, the n should refer to different individuals and as such should be indicated clearly. In addition, authors should clarify that the same individual was not considered twice as per different vaccination campaign. On the other hand, if data is available for the same individual across different vaccination campaigns authors may consider to present these results and whether the same results were obtained.

> **The revised text contains a definition of the term 'vaccination event' (p. 3), a phrase we use to describe an individual receiving the vaccine in a given season, and providing serum samples on the day of vaccination and 3-4 weeks later. We also revised Figure 1 to include graphics illustrating the data structure, i.e. the relationship between participants (individuals) and recorded vaccination events as well as prior vaccination status. We clarify in the text that the same individual may have participated several times in the study which was then recorded as separate vaccination events (p. 5). We discuss below that repeat participation in the study did not impact the results.**

2-. The fact that nine variables are analyzed questions whether the study has enough power calculation in each of the subgroups. This reviewer remains unconvinced that there is not enough number of subjects in each of the subgroups to draw any meaningful model and conclusions thereof.

> **The revised manuscript contains the power calculation (p. 11) which demonstrates that the data size was large enough to support the modeling we performed.**
>
> **In addition, we describe on p. 11 how the modeling included a feature selection step to select**

the smallest number of variables possible to achieve the best prediction, based on the Akaike information criterion. This step reduced the number of variables and therefore increased the power of analysis.

3-. The demography of the cohort is sensible in terms of age groups; however the lack of description of the "comorbidities" makes impossible to judge indeed this is a consistent variable across the age groups. Different comorbidities would have different effect on the variable under consideration.

In the light of the reviewer's comment, we added Appendix Table S5 and a section in the text (p. 6-7) which discusses the impact of specific comorbidities. We now explain that even the most abundant specific comorbidities (e.g. high cholesterol level) only affected a small number of participants (10%) which rendered it necessary to use an approximation across all comorbidities(i.e. to transform the information into simply presence/absence of *any* comorbidity).

In addition, we indeed find that depression and high cholesterol level affect *Seroconversion* negatively in *Adult1* and *Adult2* subpopulations respectively (p. 7). As depression also only affected a small fraction of the respective subpopulations (3%, p. 7), we would have not have enough power of analysis to include specific comorbidities in the models.

4-. Although this reviewer fully appreciates this is a computer-based work, the modelling and prediction is as good as it helps to explain the reality. Therefore, this work would have required additional work to validate their findings. In other words, a prospective study to test the prediction of vaccine failure based on the modelling. Otherwise, the conclusions remain at the theoretical level.

We expanded the description as to how the predictions were evaluated. First, we trained the models on data from four of the five seasons and used the data from the remaining season (UGA4) as an independent test set that was not included in the model training. This is common practice in predictive modeling and a conservative approach. We also explained in the text (p. 10) that we chose UGA4 for evaluation as it was least biased with respect to the distribution of age and prior vaccination status amongst participants.

Second, we repeated the training/modeling, but this time randomly selecting test data from the entire dataset. As before, the test data was not used for training and only for evaluation. The results were similar with respect to evaluation of prediction accuracy. The revised manuscript describes these results in Appendix Figure S5B and in the text on p. 4-5.

Third, we tried our best to identify an independent flu vaccination study (not conducted at the University of Georgia at Athens) to test the generality of the models in a completely different dataset, generated by a different group. Using the ClincialTrials.gov database, we contacted PIs from six different flu vaccine studies with a setup similar to ours (NCT01222403; NCT00363870; NCT00988143; NCT00298233; NCT03330132; NCT03266237), but we received either no reply even after a repeated contact or the data did not include measurements of HAI titer levels or similar and was therefore not useable.

5-. The study is based on a very specific population that it is very loosely described (refer also to comment 3), making hard to evaluate the consideration of race, for example. For the age variable, the numbers seem low to make any conclusion. In addition, the authors need to include as an important limitation of their study that their conclusions only apply to a restricted cohort and may not be of global interest.

We clarify in the revised manuscript that the underlying dataset is inherently biased with respect to race distribution (p. 4).

Figure 2C shows the distribution of age within each subpopulation and the legend lists the underlying total number of datapoints (n = 358, 770, and 240, for *Children*, *Adult1*, and *Adult2*, respectively). Figure 3B shows the age distributions in their relationship to BMI, again listing the total number of datapoints in the legend. We argue that they are reasonably well-distributed within each subpopulation and that the numbers are high compared to related work on much smaller cohorts.

We believe that the reviewer's comment referes to Figure 5 which shows the prediction results for both *Seroconversion* and *BaselineSY*. The plots show indeed smaller numbers of datapoints, in particular for the *Adult2* subpopulation. We clarify in the figure legend that this is because the predictions were evaluated only on the independent test set (UGA4) which is ~20% of the total dataset. The model training was done on the remaining 80% of the data (UGA1-3 and UGA5) to remove any biases specific to individual datasets as much as possible.

Appendix Figure S5B shows the evaluation for the randomly chosen test sets which are consistent with the results using the UGA4 cohort but should not suffer from any biases inherent to each cohort. Therefore, we believe our results are valid.

Minor comments.

The readability of the manuscript will improve if the authors will refer not only the figure number but also the panel when describing the results.

We addressed this comment in the revised manuscript.

Reviewer #2:

Comments for the authors of the EMBO Molecular Systems Biology manuscript number MSB-2021-10724:

The authors of the EMBO Molecular Systems Biology manuscript "Evaluation of determinants of the serological response to the split-inactivated influenza vaccine", present their efforts to use previous information regarding vaccine-induced immunity as a predictor of vaccine efficacy in specific vaccine groups. To develop this model, they have used more than 1,300 vaccine events, and they measured seroconversion and hemagglutination inhibition (HAI) titers one year post-vaccination. The group took multiple factors into consideration during this evaluation, including age, BMI, sex, race, comorbidities, pre-vaccination history, and baseline HAI. Through use of regression models, they show the strongest determinants for seroconversion are prior vaccination, baseline titer, and age. Interestingly, these three criteria had a negative effect on this outcome. The authors only report a gender effect in older populations, a small effect on vaccine month that was difficult to fully evaluate due to the timing of vaccine delivery in some populations (like the Adult 2 population), and an unexpected minimal impact for BMI (which may be due to the association between BMI and age). Overall, this is an interesting study that uses modeling to predict issues in influenza vaccination that need to be understood at both the basic science and the clinical level. Specifically, understanding the impact of predictive data on clinical outcomes, will be important for driving future vaccination campaigns. The data presented here move closer to this understanding, and I have a few comments listed below that I would like the authors to address during the revision process.

Comments:

1. I greatly appreciated the training of the models using previously collected data (4 seasons) and the prediction developed for the 5th season. While the study focuses on seroconversion results, I was wondering if there was any way to include data regarding influenza infection diagnosis in these individuals. While seroconversion may be affected, the clinical impact is worth adding to the outcomes reported.

We agree that data on vaccine efficacy with respect to subsequent flu infections would be highly relevant, but it was unfortunately not available for the UGA1-4 cohorts. We obtained this information for a very small number of participants of UGA5 (n=17) and show that indeed, individuals with high HAI titer levels at day 28 post vaccination were less likely to be infected by influenza than individuals with low titer levels (p=0.002, Appendix Table S6). This result supports the use of seroconversion which incorporates the day 0 and day 28 HAI titer levels. The revised manuscript mentions this result on p. 10.

2. I would like to learn a little more about how the authors plan to implement these findings into widespread application in the clinical setting. Would application of these findings into the clinical setting require every vaccine recipient to have their HAI tested prior to vaccination? Could there be future recommendations to provide a second vaccine in the older population that would be delivered later in the season (Jan/Feb)?

We added Appendix Figure S4 to show the prediction results when leaving baseline titer level out. We found that prediction accuracy remained largely unchanged although R^2 dropped. We discussed this result on p. 5 in the context of clinical applications that would not require a blood draw prior to receiving the vaccine.

In addition, we added a section in the Discussion on p. 9 that describes other potential clinical implications, e.g. with respect to the effect of high-dose vaccines or a booster shot later in the season. We emphasize that these should be considered very carefully. For example, the positive effect of later vaccination might be confounded by concurrent influenza infections, as we now discuss on p. 6 and p. 9.

Reviewer #3:

This is an interesting manuscript with work describing the serological response to influenza vaccination but I found the flow of the manuscript difficult to follow. This might be that I am less familiar with the approaches adopted to the analysis of the data and the way the results are presented, but I feel that the quality of the manuscript could be improved with some re-working of how it is to be presented.

We reworked the manuscript to improve the overall flow and accessibility to the reader not familiar with the modeling. For example, we completely changed Figure 1 to illustrate the data structure and terminology we used. We also edited Figure 4 illustrating the modeling workflow to clarify the different components of the analyses. We hope that these changes improved the readability.

One of the problems I had was with what was an observed result and what was predicted in a test set result

and how this was compared with the observed result in the test case. It was also not clear to me what were the models used precisely.

We have reworked Figure 4 to clarify the relationship between training sets (using observations made in UGA1-3 and UGA5) and test set (UGA4). We clarified that the evaluation examines the relationship between values observed in the test set (UGA4) and predicted for the test set (UGA4).

We also clarified that we built models for each subpopulation and each modeling goal separately. Appendix Table S1 lists these models.

I also think that improvements to the figure legends would greatly clarify what is being shown.

We have revised all figure and table legends to improve clarity.

Figure 1. I did not know what the Y-axis represented in the bar charts in panel A.

We revised Figure 1 to clarify the data structure used in the analysis. The original Figure 1A is not present in the revised manuscript.

Figure 2. I was not sure what the Y-axis in the composite baseline (log10) represented. The title itself is not appropriate - figure titles are not usually conclusions (with jargon).

We revised all figure titles to avoid conclusions in the figure title.

The new Figure 1B explains (in addition to the Methods) what we define as the composite baseline. We also included these definitions in the respective figure legends.

Figure 4. The units for predicted and observed seroconversion and BaselineSY should be fully defined.

We explain in the legend for Figure 1 that Seroconversion is unit-less (as it is a ratio of two titer levels). BaselineSY refers to HAI titer levels. We expanded axis labels on all figures to provide as much information as possible.

Figure 5. The shades of color were difficult to see on my printout for this figure and some of the others.

As the colors provide only rough guides with respect to the extent and direction of the contribution of the variables, we added numbers to each cell. Appendix Tables 2 and 3 show the data underlying the figures, as we now also explain in the figure legends.

I think the details of the precise vaccines used should be presented as supplementary materials, noting that in some cases the high dose vaccine was given (P8, second paragraph). The composition of each vaccine delivered should be given.

We added details of the vaccine to Figure 1A and to the Methods section (p. 9-10).

In addition, I did not understand whether when a vaccine recommendation was made, whether the day 0 titre would have been assessed against the previous vaccine as well as the current vaccine that was

delivered at day 0.

> **For each cohort (UGA1-5), i.e. during each season, day 0 and day 21/28 titer levels were measured against the vaccine strain administered at day 0. However, for some cohorts we also have HAI titer measurements against other strains, e.g. those used in vaccines in other seasons.**

The choice of year four (UGA 4, 2019-2020) of the study is interesting and the basis for this rather than UGA 5 (2020-2021) needs some discussion. The authors might then point out that UGA 4 2019-2020 would have been vaccinated with a 3C.3a H3N2 virus, the first time since 2015-2016.

> **The revised manuscript describes on p. 10 the reason behind choosing UGA4 for evaluation: it was the least biased of the 5 cohorts. UGA5 was biased with respect to an overrepresentation of returning participants as well as confounding effects due to the SARS-CoV2 pandemic.**
>
> **The revised manuscript also contains an extensive discussion of the fact that a new strain was used for the A/H3N2 in UGA4. This fact might explain the comparatively lower contribution of prior vaccination with respect to the A/H3N2 part of the vaccine to overall prediction accuracy, as shown in Appendix Figure . This discussion is included on p. 5 (Results section).**
>
> **We also added a section on the generalization used in the modeling that does not account for the specific strain used in each season (p. 5). We describe that this generalization is valid as there is a substantial rise in HAI titer levels even against strains that are not the respective vaccine strains (Appendix Figure S7).**

The authors might address whether retrospective data contribute to the predictions. Thus in UGA5, does a titre raised against B/Washington/02/2019 in 2020 predict a titre retrospectively against B/Colorado/06/2017 in 2019? Likewise, does a titre raised against B/Brisbane/60/2008 predict a titre raised against in a subsequent year against B/Colorado/06/2017.

> **We would first like to clarify that we use the word 'prediction' here to describe the use of our computational method to estimate the expected *Seroconversion* (or *BaselineSY*) given demographic factors, prior vaccination history, and baseline titer levels against the vaccine strains. We did not preidct titer levels (or *Seroconversion*) against one strain from knowing titer levels (or *Seroconversion*) against another strain.**
>
> **To address the reviewer's comment, we investigated the extent of possible antibody cross-reactivity, i.e. a correlation observed between *Seroconversion* measured against the vaccine strain with *Seroconversion* measured for a different strain. Indeed, as Appendix Figure S7 shows, there is substantial correlation between *Seroconversion* measured for different strains *within* the H1N1 or H3N2 group. The revised manuscript discusses these findings on p. 5.**

It is interesting that the authors suggest for the Adult1 panel that seroconversion and BaselineSY were predicted worst. They also note that this group has the widest range of vaccination dates (Figure 1). Do these correlate? (Perhaps following infection and not vaccination).

> **We revised the discussion of the impact of vaccination dates (month of vaccination) on p. 6.**

Month of vaccination is a significant predictor of vaccine efficacy in both *Children* and the *Adult1* subpopulations (Figure 6). For that reason, it is less likely to be the reason behind the relatively low performance for the *Adult1* subpopulation.

The revised manuscript discusses that the comparatively low performance for the *Adult1* subpopulation might be due to additional factors that were not considered in the model, e.g. specific comorbidities (p. 6-7) or perhaps non-linear effects (as our model only incorporates linear relationships).

P4, paragraph six. I was not sure why it was expected that prior vaccination and high baseline levels did not predict high seroconversion. The authors should elaborate.

We edited the manuscript to clarify our reasoning (p. 3). Based on the data, vaccine recipients show higher HAI titer levels against the vaccine strain (or related strains) for up to 3 years after the vaccination event (Appendix Figure S1). In other words, there is substantial, although not complete, correlation between prior vaccination (in the year prior to receiving the vaccine) and baseline titer levels measured on day 0 before receiving the vaccine (Figure 3A). If a participant has high baseline titer levels, we observe that titer levels cannot rise much more after receiving the vaccine (day 28). As seroconversion is calculated as the ratio between the day 28 and day 0 titer levels, high day 0 levels with a modest additional increase at day 28 lead to comparatively small seroconversion.

We discuss this apparent paradox when using seroconversion as a measure of vaccine efficacy on p. 7 in the manuscript. We also discuss that, for the reason described above, future vaccine efficacy studies need to incorporate existing immunity (i.e. baseline titer levels and prior vaccination status) into account (p. 7).

P5, paragraph two. The authors note a marked effect on B/Yamagata seroconversion. The authors might ask whether this is associated with the widespread circulation of the B/Yamagata lineage viruses in 2017-2018?

The revised manuscript includes a discussion of this result on p. 6. Indeed, B/Yamagata was the dominant lineage in the 2017-2018 flu season, but B lineages still were less prevalent than A subtypes, and in three of the other four seasons, B/Yamagata was even less prevalent than B/Victoria. So the reason remains unclear to us.

1st Revision - Editorial Decision 5th Apr 2022

Thank you for sending us your revised manuscript. We have now heard back from the two reviewers who agreed to evaluate your study. As you will see, the reviewers are satisfied with the modifications made and think that the study is now suitable for publication.

Before we can formally accept your manuscript, we would ask you to address the following issues:

Reviewer #1:

The authors have provided additional information and clarifications to meet my comments and those of the other two referees. The manuscript has improved substantially and the extra information will facilitate the translation of their results and modelling by others.

Reviewer #2:

The authors have addressed all of my previous comments, and I do not have any further comments at this time.

2nd Authors' Response to Reviewers 11th Apr 2022

The authors have made all requested editorial changes.

Accepted **12th Apr 2022**

Thank you again for sending us your revised manuscript. We are now satisfied with the modifications made and I am pleased to inform you that your paper has been accepted for publication.

EMBO Press Author Checklist

Corresponding Author Name: Christine Vogel
Journal Submitted to: Molecular Systems Biology
Manuscript Number: MSB-2021-10724

USEFUL LINKS FOR COMPLETING THIS FORM
The EMBO Journal - Author Guidelines
EMBO Reports - Author Guidelines
Molecular Systems Biology - Author Guidelines
EMBO Molecular Medicine - Author Guidelines

Reporting Checklist for Life Science Articles (updated January 2022)
This checklist is adapted from Materials Design Analysis Reporting (MDAR) Checklist for Authors. MDAR establishes a minimum set of requirements in transparent reporting in the life sciences (see Statement of Task: 10.31222/osf.io/9sm4x). Please follow the journal's guidelines in preparing your manuscript.
Please note that a copy of this checklist will be published alongside your article.

Abridged guidelines for figures
1. Data
The data shown in figures should satisfy the following conditions:
- the data were obtained and processed according to the field's best practice and are presented to reflect the results of the experiments in an accurate and unbiased manner.
- ideally, figure panels should include only measurements that are directly comparable to each other and obtained with the same assay.
- plots include clearly labeled error bars for independent experiments and sample sizes. Unless justified, error bars should not be shown for technical replicates.
- if n<5, the individual data points from each experiment should be plotted. Any statistical test employed should be justified.
- Source Data should be included to report the data underlying figures according to the guidelines set out in the authorship guidelines on Data Presentation.

2. Captions
Each figure caption should contain the following information, for each panel where they are relevant:
- a specification of the experimental system investigated (eg cell line, species name).
- the assay(s) and method(s) used to carry out the reported observations and measurements.
- an explicit mention of the biological and chemical entity(ies) that are being measured.
- an explicit mention of the biological and chemical entity(ies) that are altered/varied/perturbed in a controlled manner.
- the exact sample size (n) for each experimental group/condition, given as a number, not a range;
- a description of the sample collection allowing the reader to understand whether the samples represent technical or biological replicates (including how many animals, litters, cultures, etc.).
- a statement of how many times the experiment shown was independently replicated in the laboratory.
- definitions of statistical methods and measures:
  - common tests, such as t-test (please specify whether paired vs. unpaired), simple χ2 tests, Wilcoxon and Mann-Whitney tests, can be unambiguously identified by name only, but more complex techniques should be described in the methods section;
  - are tests one-sided or two-sided?
  - are there adjustments for multiple comparisons?
  - exact statistical test results, e.g., P values = x but not P values < x;
  - definition of 'center values' as median or average;
  - definition of error bars as s.d. or s.e.m.

Please complete ALL of the questions below.
Select "Not Applicable" only when the requested information is not relevant for your study.

Materials

Newly Created Materials	Information included in the manuscript?	In which section is the information available? (Reagents and Tools Table, Materials and Methods, Figures, Data Availability Section)
New materials and reagents need to be available; do any restrictions apply?	Not Applicable	

Antibodies	Information included in the manuscript?	In which section is the information available? (Reagents and Tools Table, Materials and Methods, Figures, Data Availability Section)
For **antibodies** provide the following information:<br>- Commercial antibodies: RRID (if possible) or supplier name, catalogue number and or/clone number<br>- Non-commercial: RRID or citation	Not Applicable	

DNA and RNA sequences	Information included in the manuscript?	In which section is the information available? (Reagents and Tools Table, Materials and Methods, Figures, Data Availability Section)
**Short novel DNA or RNA including primers, probes**: provide the sequences.	Not Applicable	

Cell materials	Information included in the manuscript?	In which section is the information available? (Reagents and Tools Table, Materials and Methods, Figures, Data Availability Section)
**Cell lines:** Provide species information, strain. Provide accession number in repository **OR** supplier name, catalog number, clone number, and/**OR** RRID.	Not Applicable	
**Primary cultures:** Provide species, strain, sex of origin, genetic modification status.	Not Applicable	
Report if the cell lines were recently **authenticated** (e.g., by STR profiling) and tested for mycoplasma contamination.	Not Applicable	

Experimental animals	Information included in the manuscript?	In which section is the information available? (Reagents and Tools Table, Materials and Methods, Figures, Data Availability Section)
**Laboratory animals or Model organisms:** Provide species, strain, sex, age, genetic modification status. Provide accession number in repository **OR** supplier name, catalog number, clone number, **OR** RRID.	Not Applicable	
**Animal observed in or captured from the field:** Provide species, sex, and age where possible.	Not Applicable	
Please detail **housing and husbandry conditions**.	Not Applicable	

Plants and microbes	Information included in the manuscript?	In which section is the information available? (Reagents and Tools Table, Materials and Methods, Figures, Data Availability Section)
**Plants:** provide species and strain, ecotype and cultivar where relevant, unique accession number if available, and source (including location for collected wild specimens).	Not Applicable	
**Microbes:** provide species and strain, unique accession number if available, and source.	Not Applicable	

Human research participants	Information included in the manuscript?	In which section is the information available? (Reagents and Tools Table, Materials and Methods, Figures, Data Availability Section)
If collected and within the bounds of privacy constraints report on age, sex and gender or ethnicity for all study participants.	Not Applicable	

Core facilities	Information included in the manuscript?	In which section is the information available? (Reagents and Tools Table, Materials and Methods, Figures, Data Availability Section)
If your work benefited from core facilities, was their service mentioned in the acknowledgments section?	Not Applicable	

Design

Study protocol	Information included in the manuscript?	In which section is the information available? (Reagents and Tools Table, Materials and Methods, Figures, Data Availability Section)
If study protocol has been **pre-registered, provide DOI in the manuscript**. For clinical trials, provide the trial registration number **OR** cite DOI.	Not Applicable	
Report the **clinical trial registration number** (at ClinicalTrials.gov or equivalent), where applicable.	Not Applicable	

Laboratory protocol	Information included in the manuscript?	In which section is the information available? (Reagents and Tools Table, Materials and Methods, Figures, Data Availability Section)
Provide DOI OR other citation details if **external detailed step-by-step protocols** are available.	Not Applicable	

Experimental study design and statistics	Information included in the manuscript?	In which section is the information available? (Reagents and Tools Table, Materials and Methods, Figures, Data Availability Section)

Include a statement about **sample size** estimate even if no statistical methods were used.	Yes	Figures
Were any steps taken to minimize the effects of subjective bias when allocating animals/samples to treatment (e.g. **randomization procedure**)? If yes, have they been described?	Not Applicable	
Include a statement about **blinding** even if no blinding was done.	Not Applicable	
Describe **inclusion/exclusion criteria** if samples or animals were excluded from the analysis. Were the criteria pre-established?<br><br>If sample or data points were omitted from analysis, report if this was due to attrition or intentional exclusion and provide justification.	Not Applicable	
For every figure, are **statistical tests** justified as appropriate? Do the data meet the assumptions of the tests (e.g., normal distribution)? Describe any methods used to assess it. Is there an estimate of variation within each group of data? Is the variance similar between the groups that are being statistically compared?	Yes	Figure legends

Sample definition and in-laboratory replication	Information included in the manuscript?	In which section is the information available?<br>(Reagents and Tools Table, Materials and Methods, Figures, Data Availability Section)
In the figure legends: state number of times the experiment was **replicated** in laboratory.	Not Applicable	
In the figure legends: define whether data describe **technical or biological replicates**.	Not Applicable	

Ethics

Ethics	Information included in the manuscript?	In which section is the information available?<br>(Reagents and Tools Table, Materials and Methods, Figures, Data Availability Section)
Studies involving **human participants**: State details of **authority granting ethics approval** (IRB or equivalent committee(s), provide reference number for approval.	Yes	Methods
Studies involving **human participants**: Include a statement confirming that **informed consent** was obtained from all subjects and that the experiments conformed to the principles set out in the WMA Declaration of Helsinki and the Department of Health and Human Services Belmont Report.	Yes	Methods
Studies involving **human participants:** For publication of **patient photos**, include a statement confirming that consent to publish was obtained.	Not Applicable	
Studies involving experimental **animals**: State details of **authority granting ethics approval** (IRB or equivalent committee(s), provide reference number for approval. Include a statement of compliance with ethical regulations.	Not Applicable	
Studies involving **specimen and field samples:** State if relevant **permits** obtained, provide details of authority approving study; if none were required, explain why.	Not Applicable	

Dual Use Research of Concern (DURC)	Information included in the manuscript?	In which section is the information available?<br>(Reagents and Tools Table, Materials and Methods, Figures, Data Availability Section)
Could your study fall under dual use research restrictions? Please check biosecurity documents and list of **select agents and toxins** (CDC): https://www.selectagents.gov/sat/list.htm	Not Applicable	
If you used a select agent, is the security level of the lab appropriate and reported in the manuscript?	Not Applicable	
If a study is subject to dual use research of concern regulations, is the name of the **authority granting approval and reference number** for the regulatory approval provided in the manuscript?	Not Applicable	

Reporting

The MDAR framework recommends adoption of discipline-specific guidelines, established and endorsed through community initiatives. Journals have their own policy about requiring specific guidelines and recommendations to complement MDAR.

Adherence to community standards	Information included in the manuscript?	In which section is the information available?<br>(Reagents and Tools Table, Materials and Methods, Figures, Data Availability Section)
State if relevant guidelines or checklists (e.g., **ICMJE, MIBBI, ARRIVE, PRISMA**) have been followed or provided.	Not Applicable	
For **tumor marker prognostic studies,** we recommend that you follow the **REMARK** reporting guidelines (see link list at top right). See author guidelines, under 'Reporting Guidelines'. Please confirm you have followed these guidelines.	Not Applicable	
For **phase II and III randomized controlled trials**, please refer to the **CONSORT** flow diagram (see link list at top right) and submit the CONSORT checklist (see link list at top right) with your submission. See author guidelines, under 'Reporting Guidelines'. Please confirm you have submitted this list.	Not Applicable	

Data Availability

Data availability	Information included in the manuscript?	In which section is the information available?<br>(Reagents and Tools Table, Materials and Methods, Figures, Data Availability Section)
Have **primary datasets** been deposited according to the journal's guidelines (see 'Data Deposition' section) and the respective accession numbers provided in the Data Availability Section?	Not Applicable	
Were **human clinical and genomic datasets** deposited in a public access-controlled repository in accordance to ethical obligations to the patients and to the applicable consent agreement?	Not Applicable	
Are **computational models** that are central and integral to a study available without restrictions in a machine-readable form? Were the relevant accession numbers or links provided?	Yes	Appendix
If publicly available data were reused, provide the respective **data citations in the reference list**.	Not Applicable	
